# Autoimmune demyelination alters hypothalamic transcriptome and endocrine function

**DOI:** 10.1186/s12974-023-03006-2

**Published:** 2024-01-04

**Authors:** Jonathan J. Carver, Kristy M. Lau, Alexandra E. Puckett, Alessandro Didonna

**Affiliations:** Department of Anatomy and Cell Biology, Brody School of Medicine, East Carolina University, 600 Moye Blvd., Greenville, NC USA

**Keywords:** Multiple sclerosis, Hypothalamus, Experimental autoimmune encephalomyelitis, Endocrine system

## Abstract

The hypothalamus is a brain structure that is deputed to maintain organism homeostasis by regulating autonomic function and hormonal production as part of the neuroendocrine system. Dysfunction in hypothalamic activity results in behavioral alterations, depression, metabolic syndromes, fatigue, and infertility. Remarkably, many of these symptoms are associated with multiple sclerosis (MS), a chronic autoimmune disorder of the central nervous system (CNS) characterized by focal demyelination, immune cell infiltration into the brain parenchyma, and neurodegeneration. Furthermore, altered hormonal levels have been documented in MS patients, suggesting the putative involvement of hypothalamic deficits in MS clinical manifestations. Yet, a systematic analysis of hypothalamic function in response to neuroinflammatory stress is still lacking. To fill this gap, here we performed a longitudinal profiling of the hypothalamic transcriptome upon experimental autoimmune encephalomyelitis (EAE)—a murine disease model recapitulating key MS phenotypes at both histopathological and molecular levels. We show that changes in gene expression connected with an anti-inflammatory response start already at pre-onset and persist along EAE progression. Altered levels of hypothalamic neuropeptides were also detected, which possibly underlie homeostatic responses to stress and aberrant feeding behaviors. Last, a thorough investigation of the principal endocrine glands highlighted defects in the main steroidogenic pathways upon disease. Collectively, our findings corroborate the central role of hypothalamic dysfunction in CNS autoimmunity.

**Supplementary Information:**

The online version contains supplementary material available at 10.1186/s12974-023-03006-2.

## Background

The endocrine system is a complex network of glands and organs located throughout the body that uses hormones to coordinate cellular activity and maintain homeostasis across a broad range of physiological states. Hormonal signaling is responsible for regulating food intake, energy levels, metabolic rate, blood pressure, social behaviors, stress, motivational behavior states, and many other bodily and brain functions [1]. Principal sites of hormone synthesis and release in the endocrine system include the hypothalamus, pituitary, thyroid, thymus, pancreas, adrenals, and gonads.

The hypothalamus is a part of the diencephalon that controls autonomic and somatic functions by three main neuroendocrine pathways: the hypothalamic–pituitary–thyroid (HPT) axis, the hypothalamic–pituitary–adrenal (HPA) axis, and the hypothalamic–pituitary–gonadal (HPG) axis [2]. The hypothalamus is anatomically located next to multiple circumventricular organs such as the subfornical organ (SFO), median eminence (ME), and the organum vasculum of lamina terminalis (OVLT), where the blood–brain barrier (BBB) is fenestrated and selectively permeable to blood dissolved hormones, cytokines, and metabolites [3]. Several hypothalamic nuclei, including the ventromedial preoptic area (VMPO), arcuate nucleus (ARC), dorsomedial nucleus (DMH), lateral hypothalamus (LH), and the paraventricular nucleus (PVN), are known to be involved in regulating hunger, fever, wakefulness, energy balance, and stress responses. In addition, hypothalamic nuclei make extensive contact with the brainstem and limbic system in controlling mood, arousal, stress, sleep/wake cycling, hunger, salt/water balance, and sexual functioning [4, 5]. The hypothalamus is also a critical neuroimmune integration center and has been shown to respond to systemic inflammatory stress [6]. Immunogenic challenges are known to cause a shift in behaviors toward more depressive and anxious-like states and leads to changes in brain cytokine levels and neuroglial activation, mediated at least in part by the hypothalamus [7–9].

Multiple sclerosis (MS) is an autoimmune disease of the central nervous system (CNS) characterized by demyelination, focal CNS inflammation, extensive gliosis, and neurodegeneration [10]. Multiple reports have documented changes in hypothalamus metabolism, neurotransmission, and neuroendocrine axes functioning in MS patients [11, 12]. Consistently, MS patients are more likely to report depression, anxiety, and chronic fatigue at prevalence rates at least 2–3 times higher than the general population average [13–15]*.* Disruption of neuroendocrine signaling was also described in the MS model experimental autoimmune encephalomyelitis (EAE). A severe decrease in corticotropin-releasing hormone (CRH) hypothalamic expression was measured in early EAE [16]. A significant inverse correlation was found between hypothalamic noradrenaline and serum corticosterone at EAE peak [17]. Increased plasma levels of adrenocorticotropic hormone (ACTH) were also detected upon disease [18]. Notably, pharmacologic manipulation of neuroendocrine signaling can modulate disease phenotypes, strengthening their mechanistic connection. For instance, administration of a synthetic analog of ACTH was demonstrated to suppress the development of EAE clinical symptoms [19]. Likewise, administration of gonadotropin-releasing hormone (GnRH) reduces the severity of EAE along with increased expressing of neurofilament proteins in the spinal cord [20]. Furthermore, thyroxine (T4) therapy is able to stimulate oligodendrocyte precursor cell (OPC) differentiation and promote remyelination in EAE [21]. Last, the synthetic corticosteroid methylprednisolone is commonly used in clinical settings to treat acute MS relapses due to its immunomodulatory effects and BBB restoration [22].

Despite the large body of experimental evidence, a comprehensive model linking MS pathology to altered hypothalamic signaling is still missing. Here, to further examine the impact of neuroinflammatory challenges on hypothalamus function, we employed RNA-seq technology to systematically analyze hypothalamic transcriptome dynamics along disease progression in the myelin oligodendrocyte glycoprotein (MOG)35–55 EAE paradigm. Remarkably, we show that changes in gene expression associated with an anti-inflammatory response precede the disease onset and persist through both acute and chronic EAE stages. We also documented disturbance in key metabolic pathways in the peripheral endocrine system at disease peak along with altered blood hormonal levels. Altogether, our findings pinpoint the hypothalamus as an early target site of disease and corroborate the notion that chronic neuroinflammation alters specific genetic programs in this key regulatory region of the CNS which in turn affects the correct functioning of endocrine glands.

## Materials and methods

### Induction of EAE

Active EAE was induced following previously published procedures [23]. In brief, 8–10-week-old female C57BL/6J mice (#000664, The Jackson Laboratory) were injected subcutaneously in both flanks with a total of 100 μg of MOG35-55 peptide (EZBiolab) in complete Freund's adjuvant (CFA) containing 4 mg/mL *Mycobacterium tuberculosis* (DIFCO Laboratories). Mice also received 400 ng of pertussis toxin (LIST Biological Laboratories) intraperitoneally both immediately after immunization and 48 h later. Control, mock-injected mice were injected with everything except the MOG peptide. All experimental animals were observed daily, and clinical signs were assessed as follows: 0, no signs; 1, decreased tail tone; 2, mild monoparesis or paraparesis; 3, severe paraparesis; 4, paraplegia; 5, quadriparesis; and 6, moribund or death. Mice were housed in a specific pathogen-free (SPF) facility and all procedures were performed in compliance with experimental guidelines approved by the East Carolina University Institutional Animal Care and Use Committee (IACUC).

### RNA extraction and RT-PCR

On the day of sample collection, the hypothalamus and endocrine glands (thyroid, adrenal glands, and ovaries) were quickly dissected and immediately snap-frozen on dry ice. Total RNA was extracted using Trizol Reagent (Invitrogen), and further purified with the RNeasy Mini Kit or RNeasy Micro Kit (Qiagen). DNA contaminations were removed by on-column digestion using the RNase-free DNase Set (Qiagen). For quantitative RT-PCR (qRT-PCR), 1 µg of purified RNA was reverse transcribed using the High-Capacity cDNA Transcription Kit with random primers (Applied Biosystems). Subsequently, PCRs were carried out on 10 ng of first strand cDNA using Absolute qPCR SYBR Green Fluorescein mix and PowerTrack SYBR Green Master Mix (Applied Biosystems). Validated primers from the PrimerBank database [24] were used in each reaction (Additional file 8: Table S1). The amplification was performed using the StepOne Plus Real-Time PCR system (Applied Biosystems), with initial denaturation at 95 °C for 10 min, followed by 45 cycles of denaturation at 95 °C for 15 s and annealing/extension at 60 °C for 60 s. The ΔΔCt relative quantification method was used to analyze changed expression levels, using glyceraldehyde-3-phosphate dehydrogenase (*Gapdh)* as the reference gene. For end-point RT-PCR, 10 ng of cDNA were amplified with the GoTaq G2 Green Master Mix (Promega), with initial denaturation at 95 °C for 3 min, then 40 cycles of denaturation at 95 °C for 30 s, annealing at 60 °C for 30 s, and extension at 72 °C for 60 s followed by a final extension at 72 °C for 5 min. The PCR products were visualized on a 2% agarose gel using SYBR Safe stain (Invitrogen).

### RNA-seq analysis

Total RNA was isolated from hypothalamic tissues as previously described. RNA quality was examined using the 4200 TapeStation (Agilent Technologies) and its concentration was determined with the Qubit Fluorometer (Thermo Fisher). Stranded cDNA libraries were prepared using the TruSeq Stranded LT mRNA kit (Illumina) in accordance with the manufacturer’s protocol using the poly-adenylated RNA isolation workflow. Sequencing of paired-end reads (100 bp × 2) was performed on a NextSeq 2000 platform (Illumina) at the Brody Integrative Genomics Core. Raw sequence reads were obtained from on-instrument DRAGEN (v3.8.0). Sequence reads of each sample were pseudo-aligned to the mouse reference (mm10) and the gene transcript abundance was quantified using Kallisto (v0.48.0). Differential expressed genes between experimental conditions were identified using DESeq2 (v1.34.0) in RStudio (Build 386 with R v4.4.1). *P* values lower than 0.05 after false-discovery rate (FDR) correction were considered significant.

### Antibodies

The following antibodies were used in the study: NeuN mouse monoclonal antibody (E4M5P, Cell Signaling Technology), GFAP mouse monoclonal antibody (GA5, Cell Signaling Technology), GFAP rabbit monoclonal antibody (D1F4Q, Cell Signaling Technology), OLIG2 mouse monoclonal antibody (MABN50, Sigma), CD11b rat monoclonal antibody (M1/70, Cell Signaling Technology), IBA1 rabbit monoclonal antibody (E4O4W, Cell Signaling Technology), IFNAR1 rabbit polyclonal antibody (PA5-120652, Invitrogen), anti-rabbit IgG F(ab’)2 Fragment Alexa Fluor 555 conjugate (4413, Cell Signaling Technology), anti-mouse IgG F(ab’)2 Fragment Alexa Fluor 488 conjugate (4408, Cell Signaling Technology), and anti-rat IgG (H + L) Alexa Fluor 488 conjugate (4416, Cell Signaling Technology).

### Immunohistochemistry

Experimental mice were perfused with 4% paraformaldehyde (PFA) and dissected brain specimens were post-fixed for an additional 4 h at room temperature. Brains were washed three times in PBS, then submerged in a 30% sucrose solution and allowed to sink at 4 °C before embedding in OCT compound (Tissue-Tek) and cutting. 20-µm coronal sections were placed onto Superfrost Plus slides (Fisher Scientific), permeabilized with 0.01% Triton XT-100 in PBS and blocked with 5% normal goat serum (NGS) in PBS at room temperature for 1 h. Primary antibodies diluted either 1:100 (IFNAR1) or 1:250 (GFAP, NeuN, OLIG2, CD11b, IBA1) in 1% bovine serum album (BSA) solution were applied onto the sections overnight at 4 °C. After extensive washing, the sections were incubated with secondary antibodies diluted 1:500 in 1% BSA for 1 h at room temperature in the dark. Finally, coverslips were mounted using Vectashield medium with DAPI (Vector Laboratories) and sealed with nail polish. Prepared slides were imaged at the Brody School of Medicine Cell Analysis Core using an LSM 700 confocal microscope (Zeiss) and analyzed using ImageJ software. For quantitative fluorescence measurements, the exact same lens, pinhole, laser, and detector settings were used to image mock injected control specimens and experimental EAE tissue specimens.

### Histopathologic analyses

Experimental animals were perfused with 4% PFA, and the endocrine glands were finely dissected under a stereomicroscope (Fisher Scientific). Tissue samples were then post-fixed in 4% PFA for an additional 24 h and then processed for paraffin embedding and hematoxylin and eosin (H&E) staining at the Brody School of Medicine Histology Core. Stained sections were imaged using an AxioImager M2 microscope (Zeiss). The thyroid ratio, gonado-somatic index (GSI), and adreno-somatic index (ASI) were calculated by the following formula: Index = [mass of gland (gr)/mass of body mass (gr)] × 100%. The diameter of thyroid follicles was measured by taking the average diameter through two perpendicular axes. Three regions of the thyroid were sampled in 200 × 200-pixel squares, and all follicles within the regions of interest were measured. The average thyroid follicle diameter was recorded and reported for each mouse sample. For the adrenal gland, cross-section images were analyzed by first taking the total area of the adrenal medulla and cortex. The ratio between the area occupied by the medulla vs the cortex was then calculated. To analyze the thickness of each cortical layer, the diameter of each layer was measured across three sections and averaged. The average layer thickness per mouse is reported. For the ovary, sections were manually counted for the number of follicles at each stage, as well as atretic follicles. Ovarian follicle stages were identified by their distinctive morphological characteristics. 5 sections per mouse were sampled, and the average values are reported. Finally, tissue sections with the most corpora lutea for each mouse were visually identified and counted to approximate the number of mature ova released [25].

### TUNEL assays

To examine the levels of cell death, TUNEL assays were carried out using the In Situ Cell Death Detection Kit TMR red (Roche). In brief, brain sections were permeabilized with 0.1% Triton X-100 in 0.1% sodium citrate, and then incubated with the TUNEL reaction mixture for 1 h at 37 °C. Brain sections digested with DNase I for 10 min at room temperature served as positive controls, while tissue sections incubated with labeling solution minus the terminal transferase enzyme were used as negative controls. All sections were imaged at the Brody School of Medicine Cell Analysis Core using an LSM 700 confocal microscope (Zeiss).

### Cytokine paneling

Dissected hypothalamic tissues were homogenized in lysis buffer (50 mM Tris HCl pH 7.5, 150 mM NaCl, 1% Triton XT-100) supplemented with protease inhibitors (Roche) and spun at 5000×*g* at 4 °C for 10 min. Supernatants were collected and their protein concentrations were adjusted using the Bradford assay (Invitrogen). The multiplexing analysis was outsourced at Eve Technologies Corp. using the Luminex 200 system. Forty-five markers were simultaneously measured in the samples using Eve Technologies' Mouse Cytokine 45-Plex Discovery Assay which consists of two separate kits (Millipore). The 32-plex kit detects the levels of Eotaxin, G-CSF, GM-CSF, IFNγ, IL-1α, IL-1β, IL-2, IL-3, IL-4, IL-5, IL-6, IL-7, IL-9, IL-10, IL-12(p40), IL-12(p70), IL-13, IL-15, IL-17, IP-10, KC, LIF, LIX, MCP-1, M-CSF, MIG, MIP-1α, MIP-1β, MIP-2, RANTES, TNFα, and VEGF. The 13-plex kit detects the levels of 6Ckine/Exodus2, Erythropoietin, Fractalkine, IFNβ-1, IL-11, IL-16, IL-20, MCP-5, MDC, MIP-3α, MIP-3β, TARC, and TIMP-1.

### ELISA assays

For serum collection, a terminal blood draw was taken from euthanized mice in the same estrus cycle stage (follicular phase) via cardiac puncture in the mid-morning. The collected blood was allowed to coagulate for 30 min at room temperature, before being spun at 2000×*g* at 4 °C for 10 min. The isolated serum fractions were subsequently tested for the levels of adrenocorticotropic hormone (ACTH), follicle-stimulating hormone (FSH) corticosterone (CORT), luteinizing hormone (LH), and 17β-estradiol (E2), using commercially available ELISA kits (AFG Bioscience and Biomatik Corporation). For all ELISA assays, absorbance values were measured at 450 nm using an accuSkan FC microplate reader (Fisher Scientific).

### Bioinformatic analyses

Hierarchical clustering of RNA-seq normalized counts was performed on the web-server Heatmapper, using “Average Linkage” and “Euclidean distance” as clustering method and distance measurement method [26]. Gene ontology (GO) enrichment analysis was carried out using Metascape, a web-based portal which provides comprehensive gene annotation based on GO processes, KEGG pathways, and the Reactome gene set. [27]. The portal also displays protein interaction networks based on the STRING, BioGrid, OmniPath, and InWeb_IM databases. For networks containing between 3 and 500 proteins, the Molecular Complex Detection (MCODE) algorithm is applied to identify densely connected network components.

### Statistical analyses

Differences between the means of two experimental groups were assessed with two-tailed Mann–Whitney *U*-test or with Student’s *t*-test on log-transformed data. *P* values equal to 0.05 or less were considered significant. Data were expressed as mean ± standard error of the mean (SEM). All tests were conducted with GraphPad Prism 8 software.

## Results

### Autoimmune neuroinflammation alters the hypothalamic transcriptome

**Fig. 1:**
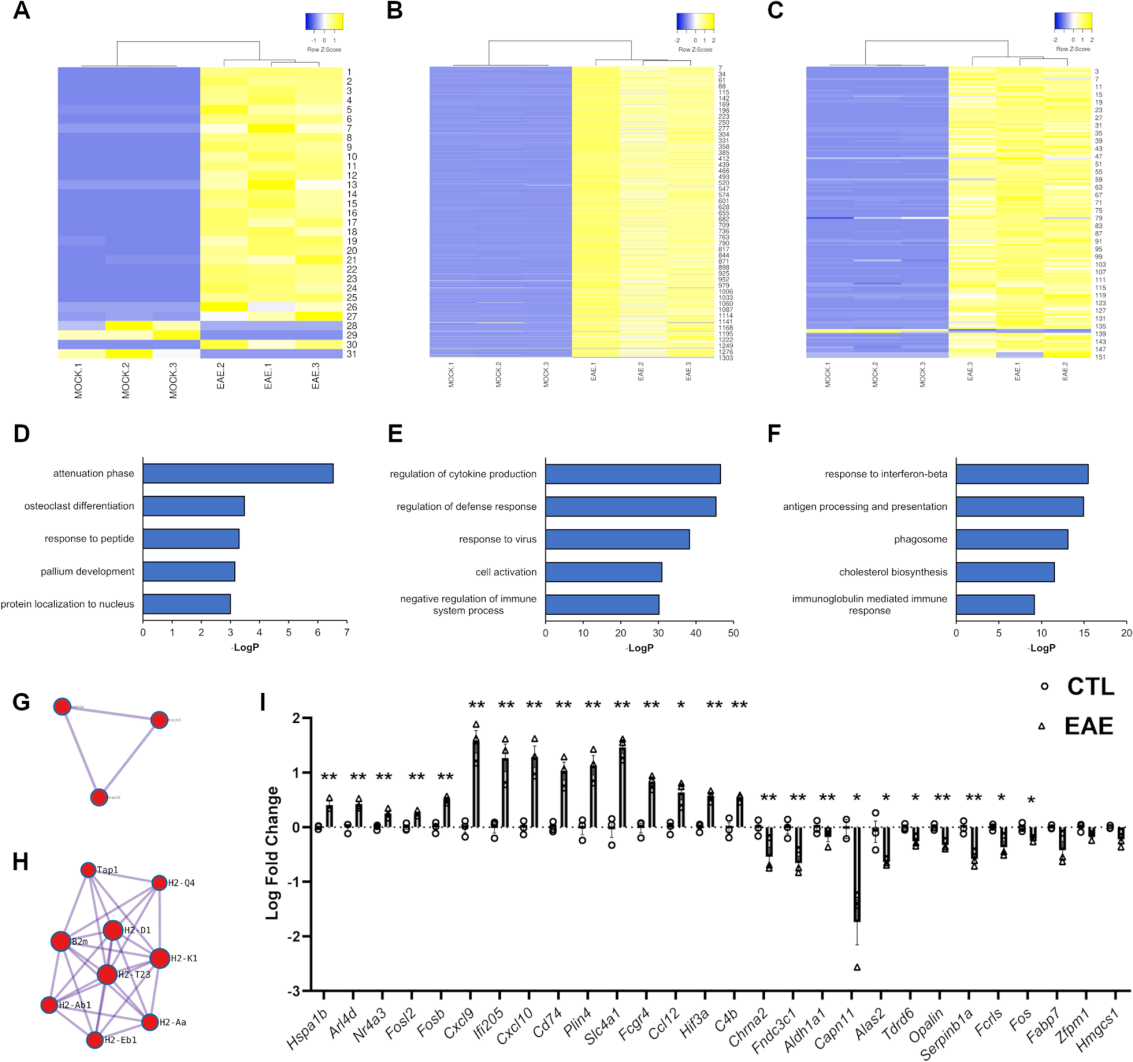
Transcriptomic profiling of the hypothalamus along EAE progression. **A**–**C** Heatmaps of normalized counts (Z-scores) for the significant differentially expressed genes (DEGs) in the hypothalamus between EAE and control mice at 10, 20, and 40 days post-immunization (dpi). Hierarchical clustering can differentiate the two experimental conditions in each cross-sectional comparison. In the heatmaps, each row represents a DEG and each column a sample. **D**–**F** Histograms showing the top 5 most enriched gene ontology (GO) terms among the significant DEGs at the different time points. **G**, **H** Most significant protein interaction MCODE networks associated with the DEGs at 10 and 40 dpi. **I** Validation of RNA-seq results by qRT-PCR on selected DEGs from each time point. All genes except *Fabp7*, *Zfpm1* and *Hmgcs1* show significant differences between EAE and control conditions. The data are expressed as log2 transformed fold change (log2FC) and plotted as means ± SEM (*N* = 3 per group from one EAE immunization). Differences between experimental groups were assessed by two-tailed Student’s *t*-test. *P ≤ 0.05, **P ≤ 0.01; ***P ≤ 0.005

To dissect the effects of neuroinflammatory stress on hypothalamus functioning, the EAE paradigm was employed in combination with next-generation sequencing. In specific, hypothalamic transcriptomes were profiled via RNA-seq technology at key EAE stages, namely baseline (0 day post-immunization, dpi), pre-onset (10 dpi), disease peak (20 dpi), and chronic phase (40 dpi) (Additional file 1: Figure S1). Cross-sectional comparison between EAE and control animals highlighted 31 differentially expressed genes (DEGs) in the hypothalamus at 10 dpi, while 1303 and 151 DEGs were respectively found at 20 and 40 dpi (Additional file 9: Table S2). Such differences were sufficient to segregate the two experimental conditions at all time-points by unsupervised hierarchical clustering (Fig. 1A–C). Subsequently, we performed gene ontology (GO) enrichment analysis to capture the molecular functions associated with these genetic signatures. The category “attenuation phase” is the most enriched ontology at pre-onset (fold increase: 220, *P* = 2.98 × 10^–7^). Conversely, the term “regulation of cytokine production” was the top ontology at 20 dpi (fold increase: 3.5, *P* = 3.23 × 10^–47^) and the term “response to interferon-beta” was the most enriched category at 40 dpi (fold increase: 30, *P* = 3.32 × 10^–16^) (Fig. 1D–F, Additional file 10: Table S3). The transcriptomic signatures at baseline and 40 dpi, but not the DEGs at disease peak, are also enriched in physically interacting gene networks as suggested by MCODE analysis (Fig. 1G-H). Importantly, we were able to independently validate by qRT-PCR 25 candidate genes among 28 randomly selected DEGs (Fig. 1I), thus confirming the accuracy and robustness of our RNA-seq data. For the 10 dpi time point, we tested heath shock protein family A member 1B (*Hspa1b*, *P* = 0.0041), ADP-ribosylation factor 4D (*Arl4d*, *P* = 0.0048), nuclear receptor subfamily 4 group A member 3 (*Nr4a3*, *P* = 0.0123), FOS like 2 (*Fosl2*, *P* = 0.0094), FosB proto-oncogene (*Fosb*, *P* = 0.0017), cholinergic receptor nicotinic alpha 2 subunit (*Chrna2*, *P* = 0.0558), fibronectin type III domain containing 3C1 (*Fndc3c1*, *P* = 0.0174), and aldehyde dehydrogenase 1 family member A1 (*Aldh1a1, P* = 0.2572); for the 20 dpi time point, we tested C-X-C motif chemokine ligand 9 (*Cxcl9*, *P* = 0.0021), C-X-C motif chemokine ligand 10 (*Cxcl10*, *P* = 0.0035), interferon activated gene 205 (*Ifi205*, P = 0.0086), cluster of differentiation 74 (*Cd74*, *P* = 0.004), perilipin 4 (*Plin4*, *P* = 0.0053), calpain 11 (*Capn11*, *P* = 0.0528), 5'-aminolevulinate synthase 2 (*Alas2*, *P* = 0.0503), tudor domain containing 6 (*Tdrd6*, *P* = 0.0161), oligodendrocytic myelin paranodal and inner loop protein (*Opalin*, *P* = 0.0048), and serine peptidase inhibitor clade B member 1a (*Serpinb1a, P* = 0.0058); for the 40 dpi time point, we tested solute carrier family 4 member 1 (*Slc4a1*, *P* = 0.0013), Fc receptor IgG low affinity IV (*Fcgr4*, *P* = 0.0019), C–C motif chemokine ligand 12 (*Ccl12*, *P* = 0.0135), hypoxia inducible factor 3 subunit alpha (*Hif3a*, *P* = 0.0016), complement C4B (*C4b*, *P* = 0.0073), Fc receptor like 2 (*Fcrls*, *P* = 0.0358), FBJ osteosarcoma oncogene (*Fos*, *P* = 0.0168), fatty acid binding protein 7 (*Fabp7*, *P* = 0.0604), zinc finger protein, multitype 1 (*Zfpm1*, *P* = 0.0649), and 3-hydroxy-3-methylglutaryl-Coenzyme A synthase 1 (*Hmgcs1, P* = 0.0607).

In parallel to cross-sectional comparisons, a longitudinal analysis across all time points was also performed to detect hypothalamic genes dynamically regulated along disease progression. This additional comparison resulted in 349 DEGs between EAE and control datasets (Additional files 2, 9: Fig. S2A, Table S2), and “regulation of defense response” was the top GO term associated with these genes (fold increase: 7, *P* = 2.82 × 10^–38^) (Additional file 2: Fig. S2B).

### The interferon-beta pathway is activated in the hypothalamus upon EAE

**Fig. 2:**
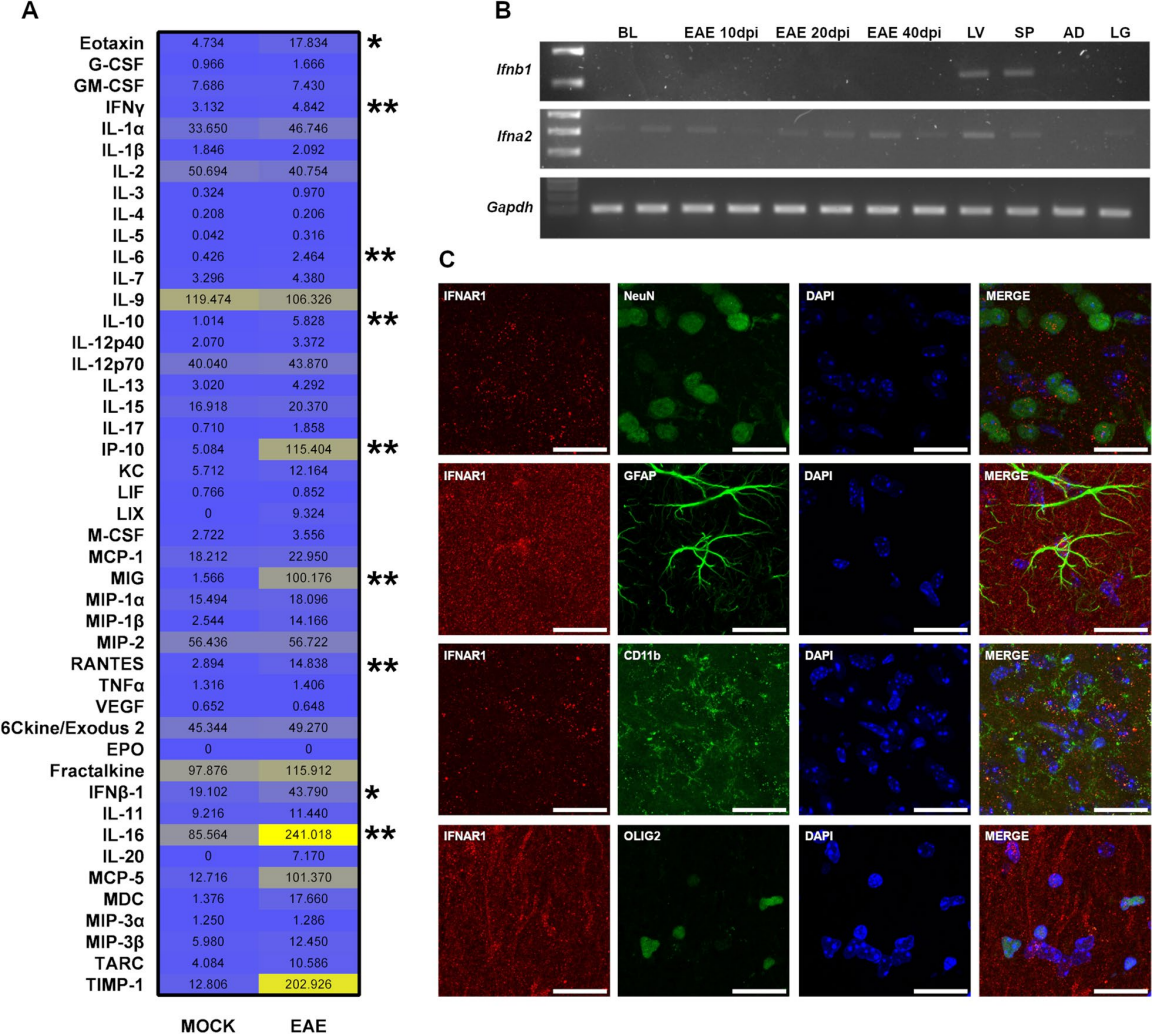
Interferon beta signaling in the hypothalamus upon EAE. **A** Heatmap of the levels of 45 cytokines in the hypothalamus of EAE and control mice at 20 dpi. Each cell represents the average concentration value expressed in pg/mL. **B** RT-PCR analysis of *Ifnb1* and *Ifna2* expression in the hypothalamus upon EAE induction. While *Ifna2* was detected at all time points, no *Infnb1* transcript was identified in the EAE hypothalamus. *Gapdh* was used as an internal control. Liver (LV), spleen (SP), adipose (AD) and lung (LG) tissues were used as additional controls. (C) Confocal analysis of co-localization patterns between IFNAR1 (red) and cell-specific markers (green) for neurons (NeuN), astrocytes (GFAP), microglia (CD11b), and oligodendrocytes (OLIG2). Nuclei were counterstained with DAPI (blue). IFNAR1 is expressed in all main cytotypes of the hypothalamus. Differences between experimental groups (*N* = 5 per group from one EAE immunization) were assessed by Mann–Whitney *U*-test. Magnification = 80 ×, scale bar = 25 μm. *P ≤ 0.05, **P ≤ 0.01; ***P ≤ 0.005

GO analysis consistently identified the interferon-beta (IFN-β) cascade as one of the top 10 enriched pathways at all the time points investigated in our transcriptomic effort. While the immunomodulatory activity of IFN-β on peripheral immune responses is well-established, much less is known about its role in CNS autoimmunity, especially in a brain region understudied as the hypothalamus. Thus, we were keen to further characterize the involvement of interferon-dependent signaling in the hypothalamic response to neuroinflammation. First, we performed a comprehensive characterization via Luminex technology of the hypothalamic cytokine profile at disease peak (20 dpi), a time point associated with the maximum DEG number. Remarkably, among the 45 cytokines tested, IFN-β levels were significantly increased in EAE mice (*P* = 0.0159) along with interferon-gamma (IFN-γ, *P* = 0.0079), interleukin-6 (IL-6, *P* = 0.0079), interleukin-10 (IL-10, *P* = 0.0079), interleukin-16 (IL-16, *P* = 0.0079), interferon gamma-induced protein 10 (IP-10, *P* = 0.0079), eotaxin (*P* = 0.0317), monokine induced by gamma (MIG, *P* = 0.0079), and regulated upon activation, normal T cell expressed and presumably secreted (RANTES, *P* = 0.0079) (Fig. 2A, Additional File 3: Fig. S3A, F). Next, we asked whether the increased IFN-β levels were the result of local synthesis of the protein or if IFN-β was rather produced in other anatomical districts. To discriminate between these two alternatives, we probed *Ifnb1* mRNA levels in the hypothalamus by RT-PCR but no detectable amounts were found at all EAE time points (Fig. 2B), which is in agreement with our RNA-seq data and corroborates the hypothesis that the hypothalamus likely represents a target site for long-range IFN-β signaling. This scenario is also consistent with the lack of astrocyte and microglia activation that were found in the EAE hypothalamus via staining for glial fibrillary acidic protein (GFAP, *P* = 0.6857), cluster of differentiation 11b (CD11b, *P* = 0.9999), and ionized calcium-binding adapter molecule 1 (IBA1, *P* = 0.4857) (Additional file 4: Fig. S4A, B).

Last, we explored the responsiveness to IFN-β activity by staining all the main neuronal cytotypes in the hypothalamus for the IFN-β receptor IFNAR1. We found that neurons, astrocytes, microglia, and oligodendrocytes express the receptor on their cell surfaces (Fig. 2C). Since IFN-β is able to mediate both pro-survival and pro-apoptotic effects [28], we also quantified possible neuronal cell death in the hypothalamus at disease peak. However, neuronal nuclei (NeuN) quantification showed similar levels of neurons in both EAE and control animals (*P* = 0.8857) (Additional file 5: Fig. S5A, B). Also, TUNEL assays failed to detect any appreciable amount of cell apoptosis in EAE hypothalamus samples (Additional file 5: Fig. S5C), therefore excluding major cell death events in this brain structure.

### Endocrine gland structure is selectively altered upon EAE

**Fig. 3:**
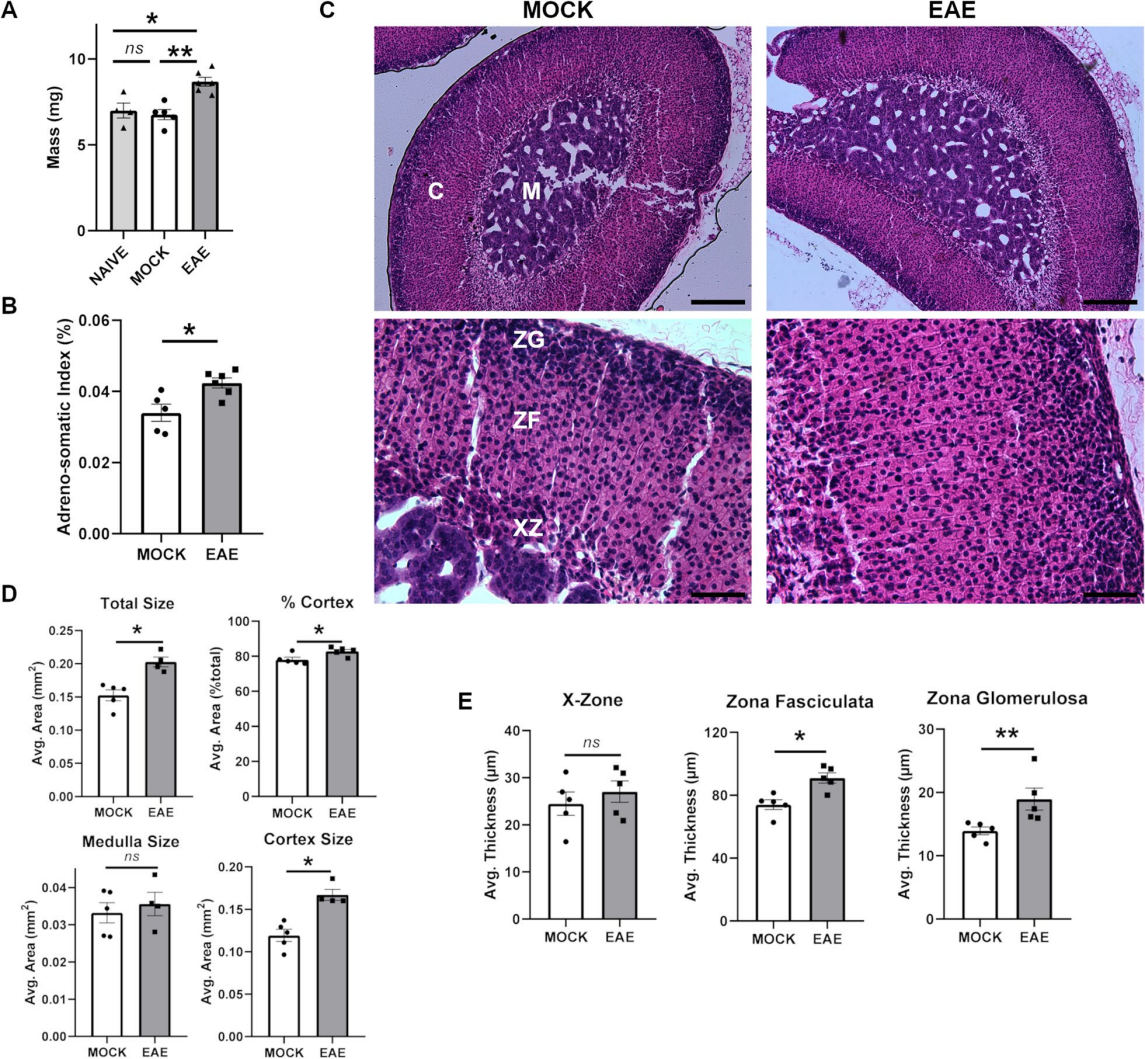
Histopathologic analysis of the adrenal glands upon EAE. **A**, **B** Bar plots showing the distributions of weight and adreno-somatic index values for adrenal glands isolated from EAE and control mice at 20 dpi. **C** Representative H&E sections at low and high magnification of adrenal glands from EAE and control mice at 20 dpi. Cortex (C) and medulla (M) are indicated in the low magnification images, while the zona glomerulosa (ZG), zona fasciculata (ZF) and X-zone (XZ) layers are identified in the high magnification images. Magnification = 10 ×, scale bar = 200 μm (top) and magnification = 40 ×, scale bar = 50 μm (bottom). **D** Quantification of the size of whole adrenal glands and sub-anatomical regions between EAE and control mice. **E** Quantification of the thickness of the different layers in adrenal glands between EAE and control mice. Differences between experimental groups (*N* = 5–6 per group from two EAE immunizations) were assessed by Mann–Whitney *U*-test. *P ≤ 0.05, **P ≤ 0.01; ***P ≤ 0.005

Fueled by our transcriptomic results, we decided to further examine the downstream effects of altered hypothalamic gene expression by analyzing the structure and function of the main endocrine glands in EAE animals at disease peak. Notably, the mass of the adrenal glands of these mice was significantly increased in both wet weight (*P* = 0.0043) and adreno-somatic index (ASI) (*P* = 0.0303) compared to mock injected and naive animals (Fig. 3A, B). Naive mice were introduced as additional controls to further exclude putative non-specific effects of the adjuvants. Upon histological inspection, the cortex but not the medulla was found responsible for the increased size of the adrenal glands upon disease (Fig. 3C, D). Within the cortex, both zona fasciculata and zona glomerulosa layers were found significantly thicker in adrenal midsections of EAE mice (*P* = 0.0159 and *P* = 0.0079 respectively) (Fig. 3E). Conversely, the size of the X-zone was not significantly different between EAE and controls (*P* = 0.5476) (Fig. 3E).

**Fig. 4:**
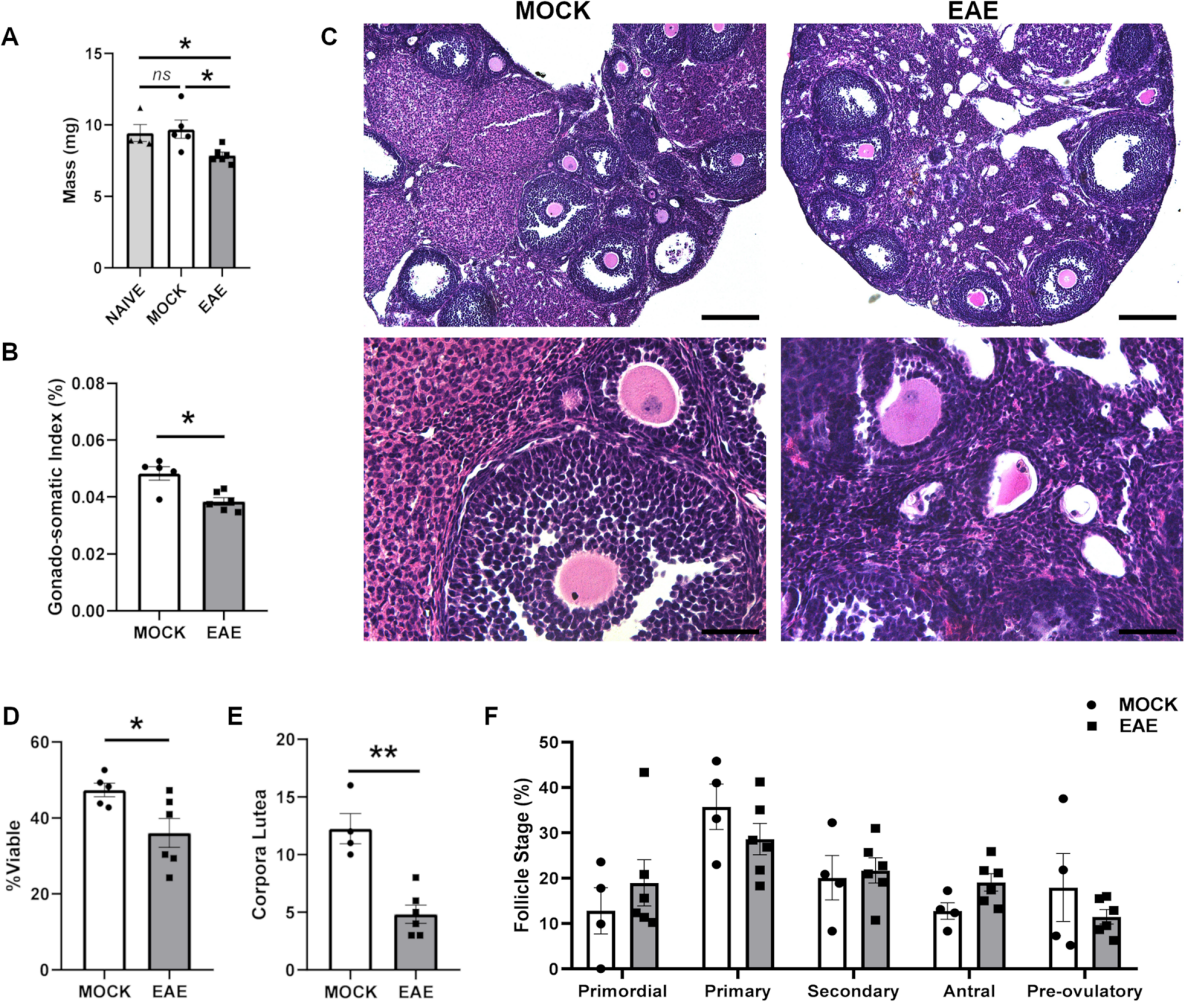
Histopathologic analysis of the ovaries upon EAE. **A**, **B** Bar plots showing the distributions of weight and gonado-somatic index values for ovaries isolated from EAE and control mice at 20 dpi. **C** Representative H&E sections at low and high magnification of ovaries from EAE and control mice at 20 dpi. Magnification = 10 ×, scale bar = 200 μm (top) and magnification = 40 ×, scale bar = 50 μm (bottom). **D**, **E** Quantification of viable follicles and corpora lutea between EAE and control mice. **F** Quantification of viable follicles within each maturation stage between EAE and control mice. Differences between experimental groups (*N* = 5–6 per group from two EAE immunizations) were assessed by Mann–Whitney *U*-test. *P ≤ 0.05, **P ≤ 0.01; ***P ≤ 0.005

In contrast with adrenal observations, the mass of the ovary in EAE mice was significantly decreased both in wet weight (*P* = 0.0108) and in gonado-somatic index (GSI) (*P* = 0.0173) compared to controls (Fig. 4A, B). During each estrus cycle, several resting primordial follicles are stimulated to develop, only a fraction of which will fully mature and ovulate. The rest undergo a specialized process of apoptotic cell death, known as follicular atresia. The percentage of viable follicles in H&E-stained sections was significantly decreased in EAE animals compared to controls (*P* = 0.0519) (Fig. 4C, D). Also, the total number of corpora lutea, both active and regressing, was significantly decreased upon EAE (*P* = 0.0095) (Fig. 4E). In contrast, the proportion of viable follicles at each stage of folliculogenesis was unchanged (Fig. 4F).

**Fig. 5:**
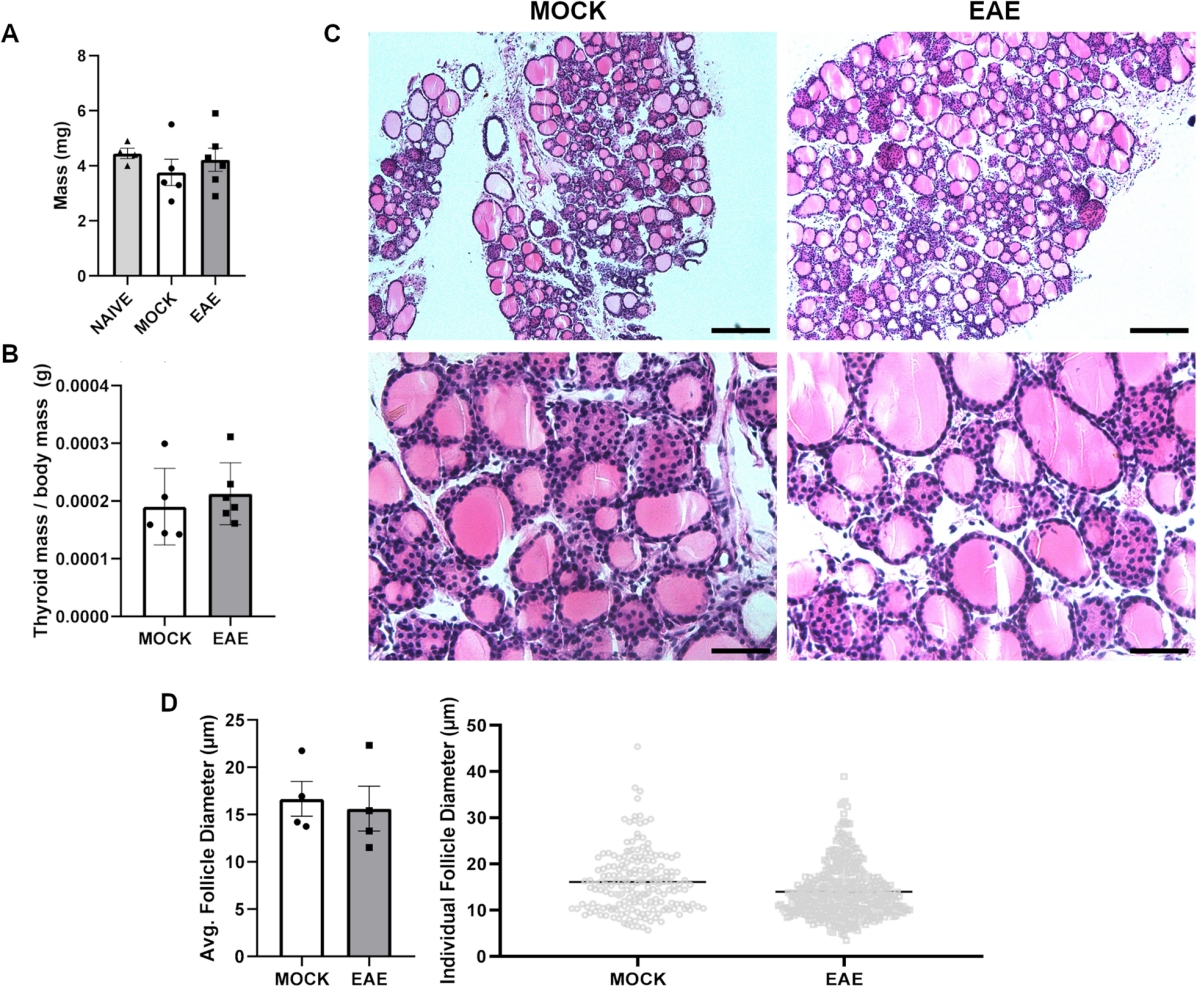
Histopathologic analysis of the thyroid upon EAE. **A**, **B** Bar plots showing the distributions of weight and thyroid-somatic index values for thyroid glands isolated from EAE and control mice at 20 dpi. **C** Representative H&E sections at low and high magnification of thyroid glands from EAE and control mice at 20 dpi. Magnification = 10 ×, scale bar = 200 μm (top) and magnification = 40 ×, scale bar = 50 μm (bottom). **D**, **E** Quantification of mean and individual follicle size between EAE and control mice. Differences between experimental groups (*N* = 5–6 per group from two EAE immunizations) were assessed by Mann–Whitney *U*-test. *P ≤ 0.05, **P ≤ 0.01; ***P ≤ 0.005

Surprisingly, no changes were documented in the thyroid glands of EAE mice either in terms of total weight (*P* = 0.3290) or proportionally to body mass (*P* = 0.3290) (Fig. 5A, B). Likewise, the average thyroid follicle size was not significantly different between EAE and control mice (*P* = 0.6857) (Fig. 5C, D).

### Specific metabolic pathways are dysregulated in the endocrine system upon EAE

**Fig. 6:**
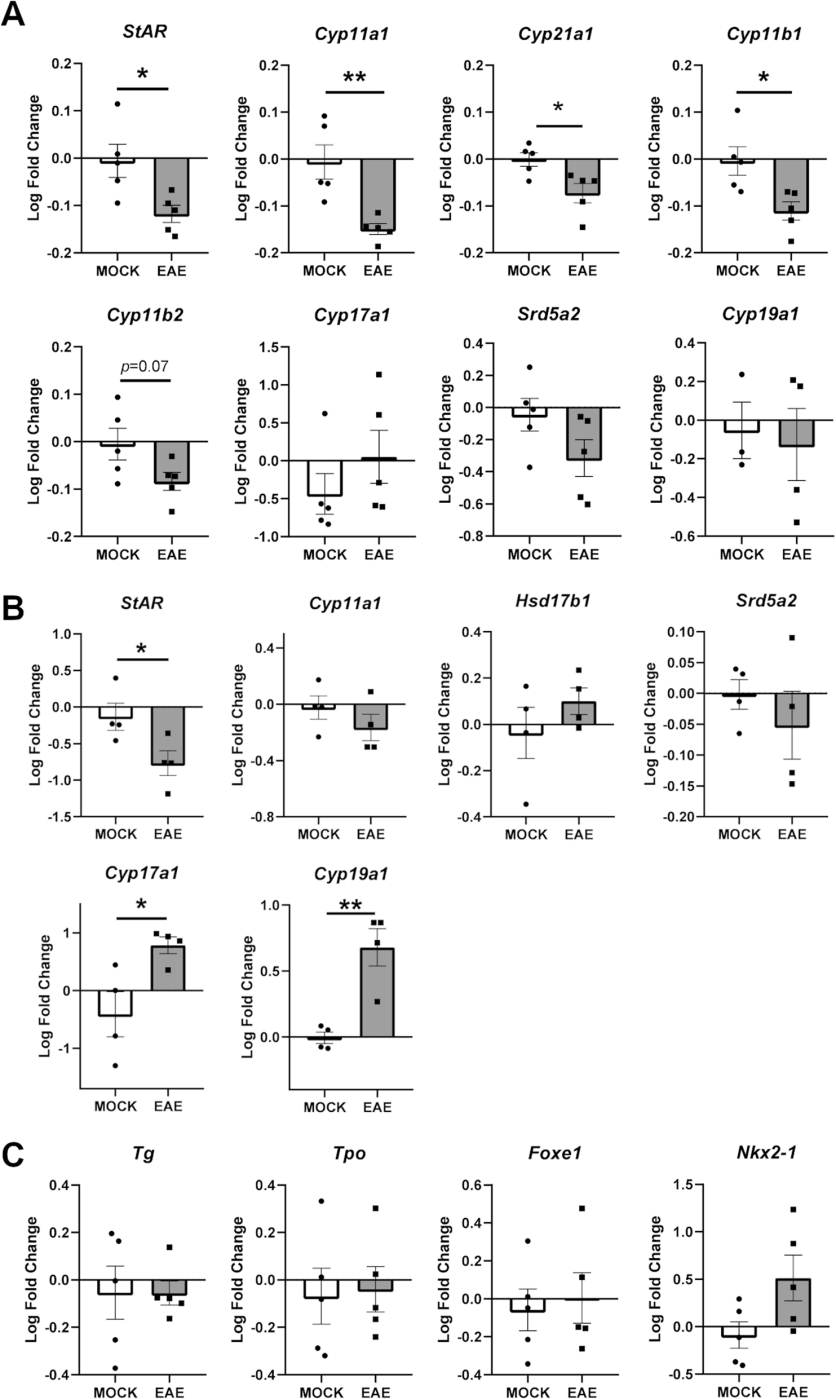
Hormonal biosynthetic pathway activity upon EAE. **A** The expression levels of key enzymes for hormonal biosynthesis were analyzed by qRT-PCR in the adrenal glands from EAE and control mice at 20 dpi. The levels of steroidogenic acute regulatory protein (*StAR*), cytochrome P450 11A1 (*Cyp11a1*) and cytochrome P450 11B1 (*Cyp11b1*) are increased in EAE mice compared to controls. No differences were found in the levels of steroid 5 alpha-reductase 2 (*Srd5a2*), cytochrome P450 17A1 (*Cyp17a1*), cytochrome P450 11B2 (*Cyp11b2*), and cytochrome P450 19A1 (*Cyp19a1*). **B** Expression analysis of the same enzymes in the ovaries. The levels of steroidogenic acute regulatory protein (*StAR*), cytochrome P450 17A1 (*Cyp17a1*) and cytochrome P450 19A1 (*Cyp19a1*) are increased in EAE mice. No differences were found in the expression levels of 17-beta dehydrogenase 1 (*Hsd17b1)*, cytochrome P450 11A1 (*Cyp11a1)*, and steroid 5 alpha-reductase 2 (*Srd5a2*). **C** Expression analysis for enzymes involved in thyroid hormonal biosynthesis. No differences were detected in the levels of hyroglobulin (*Tg*), thyroid peroxidase (*Tpo*), forkhead box protein E1 (*Foxe1*), and NK2 homeobox 1 (*Nkx2-1*). The data are expressed as log2 transformed fold change (log2FC) and plotted as means ± SEM. Differences between experimental groups (*N* = 5 per group from one EAE immunization) were assessed by two-tailed Student’s *t*-test. *P ≤ 0.05, **P ≤ 0.01; ***P ≤ 0.005

To address the possible functional consequences of the structural alterations we documented in adrenal glands and ovaries, we examined by qRT-PCR the expression at disease peak of key enzymes in the biosynthetic pathways controlling the conversion of cholesterol into the main classes of steroid hormones released by endocrine glands (Additional File 6: Fig. S6). Interestingly, despite the increased size of the adrenal gland, there was a general downregulation of steroidogenic enzymes in the adrenal cortex. Significantly downregulated enzymes include steroidogenic acute regulatory protein (*StAR, P* = 0.0216) and cytochrome P450 11A1 (*Cyp11a1*, *P* = 0.0058), which mediate the conversion of cholesterol to pregnenolone; as well as cytochrome P450 21A1 (*Cyp21a1, P* = 0.0213) and cytochrome P450 11B1 (*Cyp11b1*, *P* = 0.0191), which mediate the conversion of progesterone and 17OH-progresterone respectively to corticosterone and 11-deoxycortisol (Fig. 6A). In contrast, neither steroid 5 alpha-reductase 2 (*Srd5a2, P* = 0.1168), cytochrome P450 17A1 (*Cyp17a1, P* = 0.3007), cytochrome P450 11B2 (*Cyp11b2, P* = 0.7815) nor cytochrome P450 19A1 (*Cyp19a1, P* = 0.0755) were differentially expressed (Fig. 6A).

The main steroid products of the ovaries are estradiol during ovarian follicular phase, and progesterone during the luteal phase (Additional file 6: Fig. S6). The levels of *StAR* transcripts were significantly lower in ovaries from EAE mice (*P* = 0.0442) (Fig. 6B). Expression levels of hydroxysteroid 17-beta dehydrogenase 1 (*Hsd17b1)*, *Cyp11a1*, and *Srd5a2* were not affected in EAE animals (*P* = 0.2967, *P* = 0.3121, *P* = 0.4374 respectively) (Fig. 6B). Conversely, there was a significant increase of expression in *Cyp19a1* (*P* = 0.0036) and *Cyp17a1* (*P* = 0.0286)—the latter mediating the conversion of pregnenolone and progesterone respectively to 17OH-pregnenolone and 17OH-progresterone (Fig. 6B).

We also tested the levels of the main factors involved in the biosynthesis of thyroid hormones, namely thyroglobulin (*Tg*), thyroid peroxidase (*Tpo*), forkhead box protein E1 (*Foxe1*), and NK2 homeobox 1 (*Nkx2-1*). Paralleling the results of our histological characterization of thyroid glands, no differences in the expression of these genes were detected between EAE and control animals (Fig. 6C).

### Serum hormone levels are altered in EAE

**Fig. 7:**
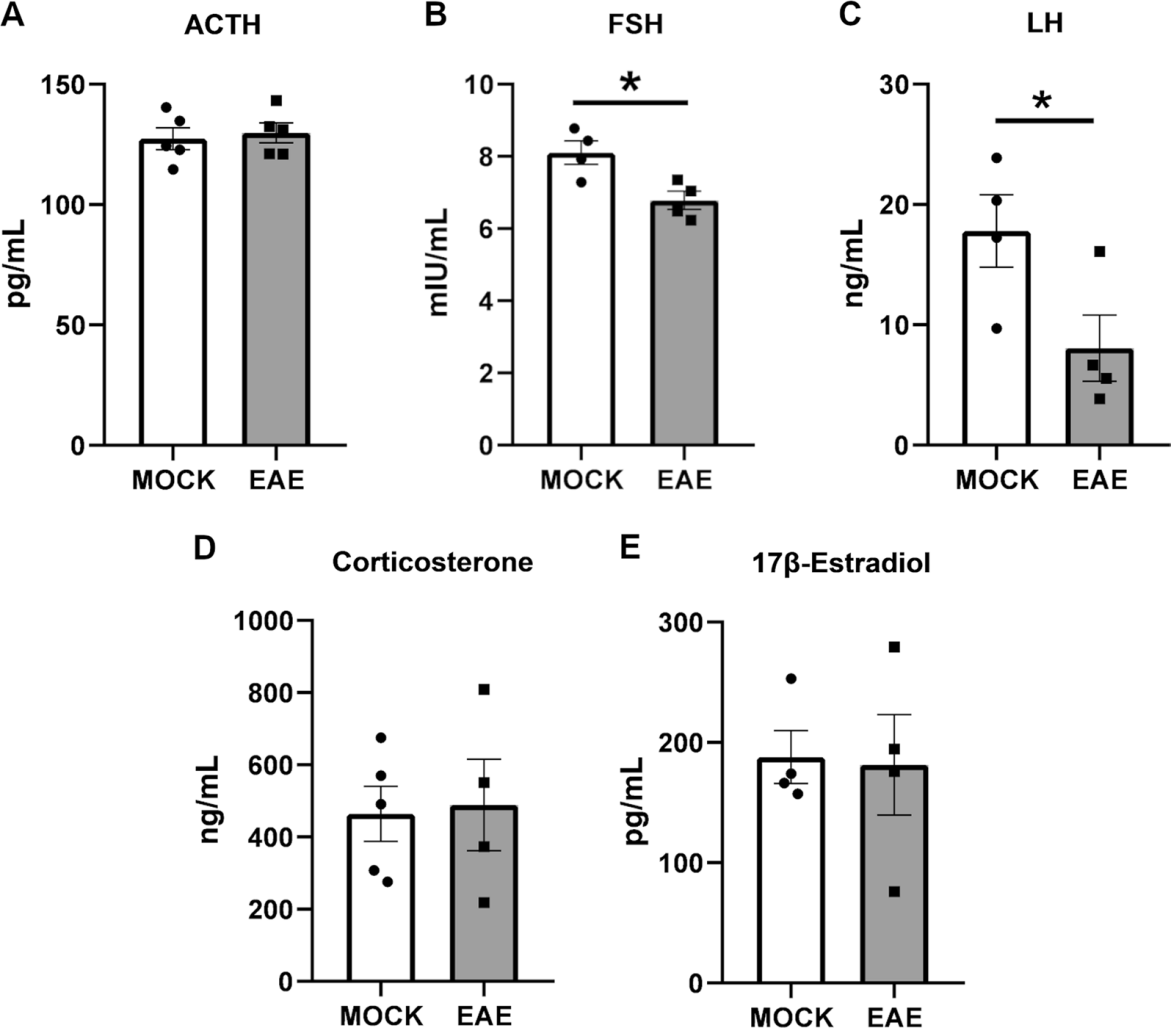
Quantification of serum hormones upon EAE. **A**–**C** Bar plots showing the concentration of the pituitary releasing-hormones adrenocorticotropic hormone (ACTH), follicle stimulating hormone (FSH), and luteinizing hormone (LH) in the serum of EAE mice and controls at 20 dpi. **D**, **E** Plots showing the serum concentration of corticosterone and estradiol at the same time points. Data are plotted as means ± SEM and differences between experimental groups (*N* = 5 per group from one EAE immunization) were assessed by Mann–Whitney *U*-test. *P ≤ 0.05, **P ≤ 0.01; ***P ≤ 0.005

A detailed quantification of circulating hormone levels upon EAE was carried out by ELISA assays, to complement our gene expression analysis on the different biosynthetic pathways. We first tested the levels of ACTH and the gonadotropic hormones follicle stimulating hormone (FSH) and luteinizing hormone (LH)—all these hormones are released from the pituitary into the circulatory system under the direct control of the hypothalamus. Serum ACTH levels were equivocal between EAE and control mice (*P* = 0.7075) (Fig. 7A). Conversely, both FSH and LH were significantly decreased in EAE mice compared with controls (*P* = 0.0185 and *P* = 0.0543 respectively) (Fig. 7B, C). The ELISA results are consistent with follicular phase levels during the estrus cycle, with higher FSH than LH in both EAE and control animals. Also, the ratios between LH/FSH were not increased between experimental conditions (*P* = 0.1488). We then tested serum corticosterone and estradiol levels, which are released respectively by adrenal glands and ovaries. Similar concentrations for both hormones were detected between EAE and control mice (*P* = 0.8683 and *P* = 0.8962 respectively) (Fig. 7D, E).

## Discussion

Significant efforts have been put toward understanding the molecular basis of MS clinical manifestations. This has led to the discovery of multiple fundamental mechanisms governing the aberrant immune response and consequent neurodegeneration occurring in MS patients. Conversely, the molecules and cytotypes mediating the psychological and metabolic symptoms that associate with the main pathological phenotypes are far from being fully elucidated. Yet, the presence of these comorbidities adds a substantial burden on MS patients’ quality of life and decreases patient adherence to prescribed medications [14]. To fill this gap, we have performed a comprehensive characterization of the hypothalamic–endocrine system in a relevant animal model of MS. Our data support a working model for disease pathogenesis in which encephalitogenic stress is sufficient to alter hypothalamic functioning in the absence of local demyelinating lesions, via aberrant cytokine signaling. This is in line with previous reports documenting disease phenotypes in other areas of the EAE brain not subjected to extensive lesion formation and immune cell infiltration [29–31]. We further show that endocrine glands display distinct responses to altered hypothalamic signaling during EAE, which may have either homeostatic or pathogenic implications **(**Additional file 7: Fig. S7).

Several lessons can be learned from our study. First, our transcriptomic profiling highlighted that extensive gene expression changes take place in the hypothalamus along EAE progression, starting before the disease onset. These findings are consistent with early reports showing gene dysregulation in the CNS of EAE mice before histological evidence of demyelination and inflammation [32]. Among the main pathways associated with the identified gene signatures, we further characterized the IFN-β cascade in terms of cytokine synthesis and cell responsiveness. Remarkably, disturbance in IFN-β signaling has been documented in MS patients and IFN-β drugs were the first disease-modifying therapies to treat the relapsing–remitting form of the disease [33]. From a physiological standpoint, IFN-β exerts an immunomodulatory function by acting on cytokine networks [34]. Consistently, we measured higher levels of the anti-inflammatory cytokine IL-10 in the hypothalamus at disease peak. This evidence suggests that IFN-β signaling likely represents a protective response to contrast inflammatory injury as demonstrated by elevated levels of pro-inflammatory cytokines we also detected in the hypothalamus (IFN-γ, IL-6, IL-16, and IP-10). IFN-β also possesses anti-viral properties [35], and the fact that ontologies such as “response to virus”, “Epstein–Barr virus infection”, and “Coronavirus disease” are enriched in our gene expression dataset pinpoints a possible molecular overlap between autoimmune demyelination and viral challenges. In this light, it is intriguing that Epstein–Barr virus (EBV) infection has been recently characterized as the main environmental factor for MS susceptibility [36].

Notably, multiple hypothalamic neuropeptides known to be involved in regulating physiology and behavior were found differentially expressed in our transcriptomic screening. Agouti-related neuropeptide (*Agrp*), neuropeptide Y (*Npy*), arginine vasopressin (*Avp*), and thyrotropin releasing hormone (*Trh*) show increased expression levels along EAE progression. On the contrary, pro-opiomelanocortin (*Pomc*) is downregulated upon disease. *Agrp* and *Npy* have opposite actions on food intake compared to *Pomc* and their trends may be explained with a feedback response to the aberrant appetitive behaviors induced by the disease [37–39]. In fact, EAE mice display significant decreases in body weight and spike in serum concentrations of the anorexigenic hormone leptin before the onset of neurological disability [40]. Elevated levels of *Npy* may also represent a homeostatic response to stress, due to its anxiolytic and neuroprotective functions [41]. This is supported by the pre-symptomatic GO enrichment in “attenuation phase” genes, which are typically expressed upon recovery from stress [42]. Augmented *Avp* transcription may be mechanistically connected with the disease since this neuropeptide regulates the BBB permeability and could facilitate immune cell infiltration and soluble factor penetration into the CNS [43]. Also, *Trh* upregulation may represent a pathogenic event as this neuropeptide stimulates the pituitary release of prolactin, a hormone found increased in EAE and involved in pro-inflammatory Th1/Th17 responses [44, 45]. Comparable neuropeptide dynamics have been recently reported in a rat model of EAE, further corroborating our findings [46].

The second main finding of our investigation is the selective effect of disease on endocrine gland functioning. In specific, we documented the hyperplasia of the adrenal gland and atrophy of the ovaries in EAE animals at disease peak. Clinical studies have demonstrated significantly enlarged adrenal glands and elevated urinary cortisol in MS patients [47, 48]. Likewise, mice at pre-symptomatic EAE also show increased corticosterone levels [49]. Chronic overstimulation of the adrenal gland by ACTH is known to lead to hyperplasia of cortical layering [50]. Consistent with this hypothesis, the decreased levels of steroidogenic enzymes we detected in the adrenal glands may represent the result of ACTH receptor resistance to chronic overstimulation. Also, the downregulation of the glucocorticoid receptor gene (*Nr3c1*) and the concomitant increase in *Avp* expression in the hypothalamus, at both acute and chronic EAE stages, are indicative of possible corticosterone resistance of CRH+ neurons in the PVN. Interestingly, HPA hypo-responsiveness seems to be required for disease susceptibility and severity. Indeed, intact adrenal glands are necessary for recovery from EAE, and adrenalectomized rats never recover but develop a fatal disease [51]. Stimulation of the HPA axis activity by dexamethasone challenges similarly confers resistance to EAE, while glucocorticoid receptor inhibition by mifepristone (RU38486) treatment increases the susceptibility to disease [52]. Dysregulation of the HPA axis has been also implicated in several human psychiatric conditions including major depressive disorder (MDD), post-traumatic stress disorder (PTSD), and bipolar disorder (BD), and may contribute to the development of dysphoric and dysthymic symptoms in MS patients [4]. Consistently, EAE mice experience significantly higher anxious and depressive-like behaviors either before the onset of neurological disability or in only mildly impaired animals, demonstrating their independence from motor dysfunction [49, 53].

Unlike the HPA axis, the data on ovarian dysfunction in MS are scarce. Some clinical evidence exists on diminished ovarian volume and follicular reserve in MS patients [54]. However, whether these phenotypes are the result of MS pathogenic process, or a side effect of immunosuppressant treatment has been a matter of controversy. Our data suggest that the disease per se is sufficient to induce ovarian atrophy, and this effect is likely driven by impaired LH and FSH signaling. The fact that ovarian atrophy is also induced in the neurotoxic cuprizone MS model suggests that demyelination is the main trigger of this phenotype [55]. However, a direct pathogenic activity of chronic inflammation on ovarian follicular dynamics cannot be ruled out. In fact, successful folliculogenesis, oocyte maturation, and ovulation require a precise and well-orchestrated inflammatory response [56]. The identified gonadal abnormalities may account, at least in part, for the large gap in fertility rates between women with MS and the general population [57].

## Conclusions

Altogether, our results show there is a hypothalamic response to chronic neuroinflammation, which precedes the disease onset and eventually leads to endocrine axes dysfunction. Both pathogenic and protective responses were highlighted, along with multiple dysregulated pathways and genes that may serve as novel therapeutic targets. However, several limitations still exist in our study. First, only females were used in our experiments, hence we were unable to capture possible sex-effects in the hypothalamic response to encephalitogenic challenges. Second, bulk RNA-seq was used for our transcriptomic profiling, which could not account for the complex cytoarchitecture of the hypothalamus. Last, it should be recognized that no EAE model can fully recapitulate the complexity of MS pathology. Thus, while our chosen EAE protocol efficiently models neuroinflammatory stress and severe chronic CNS demyelination, caveats should be applied in directly translating the animal data to human disease. In the future, it will be important to expand our analysis to EAE paradigms characterized by different clinical trajectories such as a relapsing–remitting disease course, which may help elucidating whether hypothalamic signaling is associated with the recovery phases possibly via the IFN-β pathway. Furthermore, the employment of the latest single-cell approaches in genomics research will be instrumental to tackle the cellular heterogeneity of hypothalamic function and dissect the contribution of different neuronal populations to the overall responses.

### Supplementary Information

**Additional file 1: Figure S1.** EAE clinical course and tissue collection time points. EAE was induced in 40 C57BL/6J female mice between 8-10 weeks of age via immunization with MOG35-55 peptide according to the protocol described in the Materials and Methods section. Additional 30 animals matched in age and sex were mock injected with everything but the peptide and served as controls. Mice were scored daily up to 40 days post-immunization (dpi). Hypothalamic tissues were dissected from 3 mice randomly selected from both cohorts at baseline (o dpi), before onset (10 dpi), at disease peak (20 dpi), and at chronic stages (40 dpi). Mean scores ±SEM are plotted.

**Additional file 2: Figure S2.** Longitudinal profiling of the hypothalamic transcriptome upon EAE. **A** Volcano plot of the differentially expressed genes (DEGs) in the hypothalamus along EAE progression. Each point represents the average value of 3 independent samples. **B** Histogram showing the top 5 most enriched gene ontology (GO) terms among the significant DEGs (adjusted *P *< 0.05).

**Additional file 3: Figure S3.** Dysregulated cytokines in the hypothalamus upon EAE. **A**–**I** Bar plots showing the concentration levels of interferon-gamma (IFN-γ), interleukin-6 (IL-6), interferon-beta (IFN-β), interleukin-16 (IL-16), interleukin-10 (IL-10), interferon gamma-induced protein 10 (IP-10), monokine induced by gamma (MIG), eotaxin, and regulated upon activation, normal T cell expressed and presumably secreted (RANTES) in the hypothalamus of EAE and control mice at 20 dpi. Data are plotted as means ±SEM (*N* = 5 per group from one EAE immunization) and differences between experimental groups were assessed by Mann-Whitney *U*-test. *P ≤ 0.05, **P ≤ 0.01; ***P ≤ 0.005.

**Additional file 4: Figure S4.** Analysis of reactive glia in the hypothalamus upon EAE. **A** Representative images of immunofluorescence staining for astrocytes (GFAP, red) and microglia (CD11b and IBA1, green) in the hypothalamus of EAE mice and controls at 20 dpi. Nuclei were counterstained with DAPI (blue). **B** Quantification of the different stains expressed as mean fluorescent intensity (MFI) values. Data are plotted as means ±SEM (N = 4 per group from one EAE immunization) and differences between experimental groups were assessed by Mann-Whitney U-test. Magnification = 10 ×, scale bar = 50 μm.

**Additional file 5: Figure S5.** Analysis of neurodegeneration in the hypothalamus upon EAE. **A** Representative images of TUNEL staining (red) in the hypothalamus of EAE mice and controls at 20 dpi. Nuclei were counterstained with DAPI (blue). **B** Staining and relative quantification of neuronal cells (NeuN) in the hypothalamus at the same time point. Magnification = 20 ×, scale bar = 25 μm. **C** Representative images of TUNEL stained (red) hypothalamic sections from EAE and control mice at 20 dpi. As positive control, some sections were treated with DNase I. Nuclei were counterstained with DAPI (blue). Magnification = 10 ×, scale bar = 25 μm. Data are plotted as means ± SEM (*N* = 4 per group from one EAE immunization) and differences between experimental groups were assessed by Mann-Whitney *U*-test.

**Additional file 6: Figure S6.** Target genes in the steroidogenesis pathway. A scheme of the main biosynthetic pathways for steroid hormones is depicted. Cholesterol is the common precursor for all the classes: progestins (purple), androgens (blue), estrogens (pink), glucocorticoids (red), and mineralocorticoids (orange). In female mice, corticosteroids and DHEA are mainly produced in the adrenal cortex layers. Estrogens, progestins, and some androgens are produced from ovarian follicles and corpora lutea in the ovary. Steroidogenic enzyme genes tested by qRT-PCR for differential expression between EAE and control animals are signified by a yellow star. Chemical structures were drawn using MolView software.

**Additional file 7: Figure S7.** Proposed working model of hypothalamic dysfunction in autoimmune demyelination. EAE pathology specifically leads to an increase of specific cytokines (CCL-11, IFNγ, IFNβ, IL-6, IL-10, and IL-16, MIG, IP-10, and RANTES) in in the absence of local lesions or reactive glial species in the hypothalamus. At the same time, several neuropeptides involved in maintaining the physiological homeostasis are differentially expressed, including *Agrp*, *Npy*, *Avp*, *Agt*, *Pomc*, *Trh*, glucocorticoid receptor *Nr3c1* and its cochaperone *Fkbp5*. The hypothalamus contains pulse generator neurons for the HPT/HPA axes in the paraventricular nucleus (PVN), and HPG axis in the arcuate nucleus (ARC) and preoptic area (POA). During acute EAE, the adrenal gland is significantly enlarged with decreased expression of steroidogenic enzymes, suggesting chronic overstimulation of HPA axis and gaining ACTH resistance. Conversely, the ovaries are significantly smaller with fewer viable follicles and corpora lutea, likely due to lower levels of both gonadotropins FSH and LH. The overexpression of *Cyp19a1* may represent a homeostatic response to maintain estrogen production.

**Additional file 8: Table S1.** List of primers used in qRT-PCR validation experiments.

**Additional file 9: Table S2.** List of differentially expressed genes in the hypothalamus between EAE and control mice.

**Additional file 10: Table S3.** Gene ontology (GO) analysis results on the differentially expressed genes.

## Data Availability

The datasets used and/or analyzed during the current study are available from the corresponding author on reasonable request.

## Abbreviations

ACTH: Adrenocorticotropic hormone
*Agrp*: Agouti-related neuropeptide
*Alas2*: 5′-Aminolevulinate synthase 2
*Aldh1a1*: Aldehyde dehydrogenase 1 family member A1
*Arl4d*: ADP-ribosylation factor 4D
ARC: Arcuate nucleus
ASI: Adreno-somatic index
*Avp*: Arginine vasopressin
BBB: Blood–brain barrier
BD: Bipolar disorder
BSA: Bovine serum albumin
*C4b*: Complement C4B
*Capn11*: Calpain 11
*Ccl12*: C–C motif chemokine ligand 12
CD11b: Cluster of differentiation 11b
*Cd74*: Cluster of differentiation 74
CFA: Complete Freund's adjuvant
*Chrna2*: Cholinergic receptor nicotinic alpha 2 subunit
CNS: Central nervous system
CORT: Corticosterone
CRH: Corticotropin-releasing hormone
*Cyp11a1*: Cytochrome P450 11A1
*Cyp17a1*: Cytochrome P450 17A1
*Cyp19a1*: Cytochrome P450 19A1
*Cyp21a1*: Cytochrome P450 21A1
*Cyp11b1*: Cytochrome P450 11B1
*Cyp11b2*: Cytochrome P450 11B2
*Cxcl9*: C-X-C motif chemokine ligand 9
*Cxcl10*: C-X-C motif chemokine ligand 10
DEG: Differentially expressed gene
DMH: Dorsomedial nucleus
dpi: Day post-immunization
E2: 17β-Estradiol
EAE: Experimental autoimmune encephalomyelitis
EBV: Epstein–Barr virus
*Fabp7*: Fatty acid binding protein 7
*Fcgr4*: Fc receptor IgG low affinity IV
*Fcrls*: Fc receptor like 2
FDR: False discovery rate
*Fndc3c1*: Fibronectin type III domain containing 3C1
*Fosb*: FosB proto-oncogene
*Fos*: FBJ osteosarcoma oncogene
*Fosl2*: FOS like 2
*Foxe1*: Forkhead box protein E1
FSH: Follicle-stimulating hormone
*Gapdh*: Glyceraldehyde-3-phosphate dehydrogenase
GFAP: Glial fibrillary acidic protein
GnRH: Gonadotropin-releasing hormone
GO: Gene ontology
GSI: Gonado-somatic index
H&E: Hematoxylin and eosin
*Hif3a*: Hypoxia inducible factor 3 subunit alpha
*Hmgcs1*: 3-Hydroxy-3-methylglutaryl-Coenzyme A synthase 1
HPA: Hypothalamic–pituitary–adrenal
HPG: Hypothalamic–pituitary–gonadal
HPT: Hypothalamic–pituitary–thyroid
*Hsd17b1*: Hydroxysteroid 17-beta dehydrogenase 1
*Hspa1b*: Heath shock protein family A member 1B
IBA1: Ionized calcium-binding adapter molecule 1
*Ifi205*: Interferon activated gene 205
IFN-β: Interferon-beta
IFN-γ: Interferon-gamma
IL-6: Interleukin-6
IL-10: Interleukin-10
IL-16: Interleukin-16
IP-10: Interferon gamma-induced protein 10
log2FC: Log2 transformed fold change
LH: Luteinizing hormone
LH: Lateral hypothalamus
MCODE: Molecular complex detection
MDD: Major depressive disorder
ME: Median eminence
MFI: Mean fluorescent intensity
MIG: Monokine induced by gamma
MOG: Myelin oligodendrocyte glycoprotein
MS: Multiple sclerosis
NGS: Normal goat serum
*Nkx2-1*: NK2 homeobox 1
*Npy*: Neuropeptide Y
*Nr4a3*: Nuclear receptor subfamily 4 group A member 3
*Nr3c1*: Glucocorticoid receptor gene
NeuN: Neuronal nuclei
*Opalin*: Oligodendrocytic myelin paranodal and inner loop protein
OPC: Oligodendrocyte precursor cell
OVLT: Organum vasculum of lamina terminalis
PFA: Paraformaldehyde
*Plin4*: Perilipin 4
*Pomc*: Pro-opiomelanocortin
PTSD: Post-traumatic stress disorder
PVN: Paraventricular nucleus
qRT-PCR: Quantitative RT-PCR
RANTES: Regulated upon activation, normal T cell expressed and presumably secreted
SEM: Standard error of the mean
*Serpinb1a*: Serine peptidase inhibitor clade B member 1a
SFO: Subfornical organ
*Slc4a1*: Solute carrier family 4 member 1
*Srd5a2*: Steroid 5 alpha-reductase 2
*StAR*: Steroidogenic acute regulatory protein
T4: Thyroxine
*Tg*: Thyroglobulin
*Tdrd6*: Tudor domain containing 6
*Tpo*: Thyroid peroxidase
*Trh*: Thyrotropin releasing hormone
VMPO: Ventromedial preoptic area
*Zfpm1*: Zinc finger protein, multitype 1
