# Supplementary figures and images for: Autoimmune demyelination alters hypothalamic transcriptome and endocrine function
Source: J Neuroinflammation. 2024 Jan 4;21:12. doi: 10.1186/s12974-023-03006-2 (PMC10768476; doi:10.1186/s12974-023-03006-2)

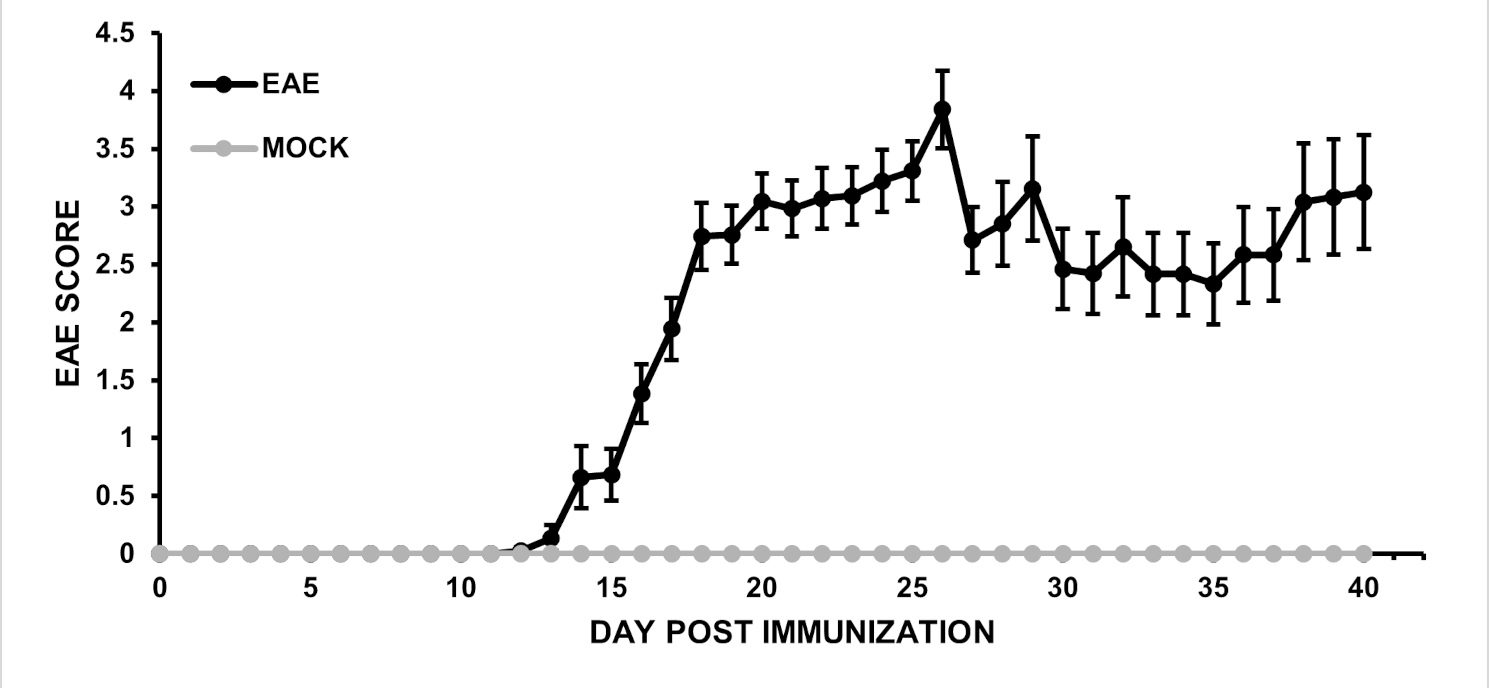

Supplement: Supplementary file 1 — Additional file 1: Figure S1. EAE clinical course and tissue collection time points. EAE was induced in 40 C57BL/6J female mice between 8-10 weeks of age via immunization with MOG35-55 peptide according to the protocol described in the Materials and Methods section. Additional 30 animals matched in age and sex were mock injected with everything but the peptide and served as controls. Mice were scored daily up to 40 days post-immunization (dpi). Hypothalamic tissues were dissected from 3 mice randomly selected from both cohorts at baseline (o dpi), before onset (10 dpi), at disease peak (20 dpi), and at chronic stages (40 dpi). Mean scores ±SEM are plotted. [file 12974_2023_3006_MOESM1_ESM.tif]

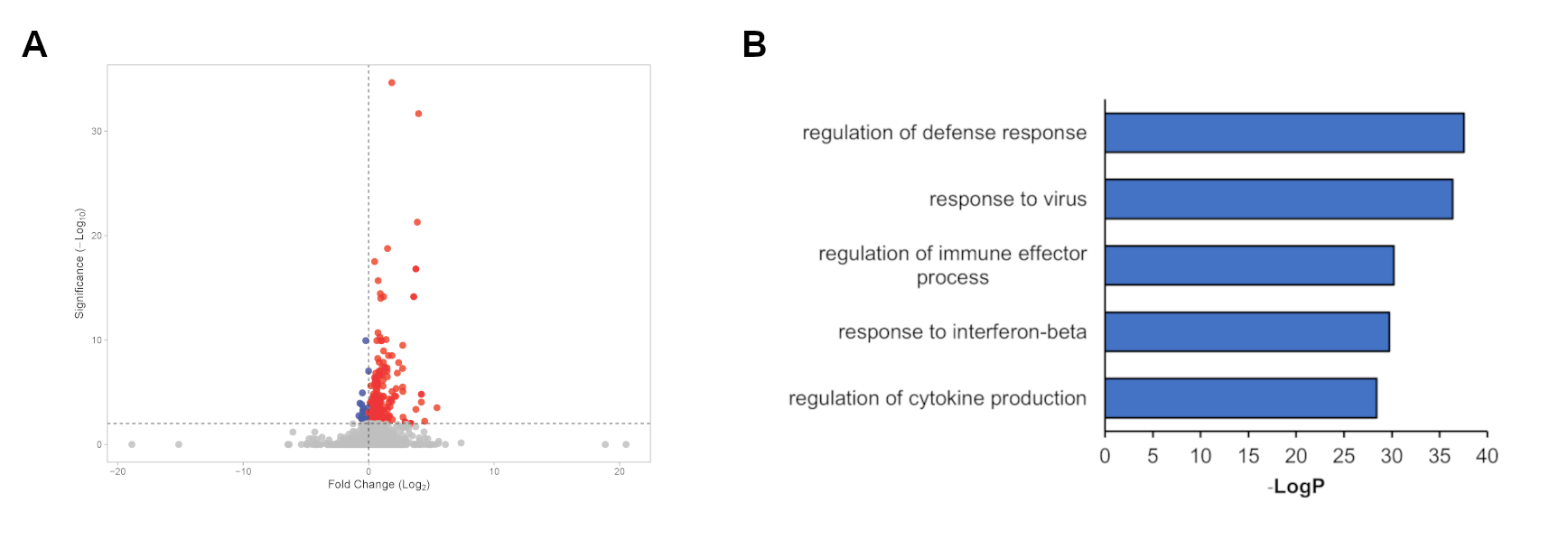

Supplement: Supplementary file 2 — Additional file 2: Figure S2. Longitudinal profiling of the hypothalamic transcriptome upon EAE. A Volcano plot of the differentially expressed genes (DEGs) in the hypothalamus along EAE progression. Each point represents the average value of 3 independent samples. B Histogram showing the top 5 most enriched gene ontology (GO) terms among the significant DEGs (adjusted P < 0.05). [file 12974_2023_3006_MOESM2_ESM.tif]

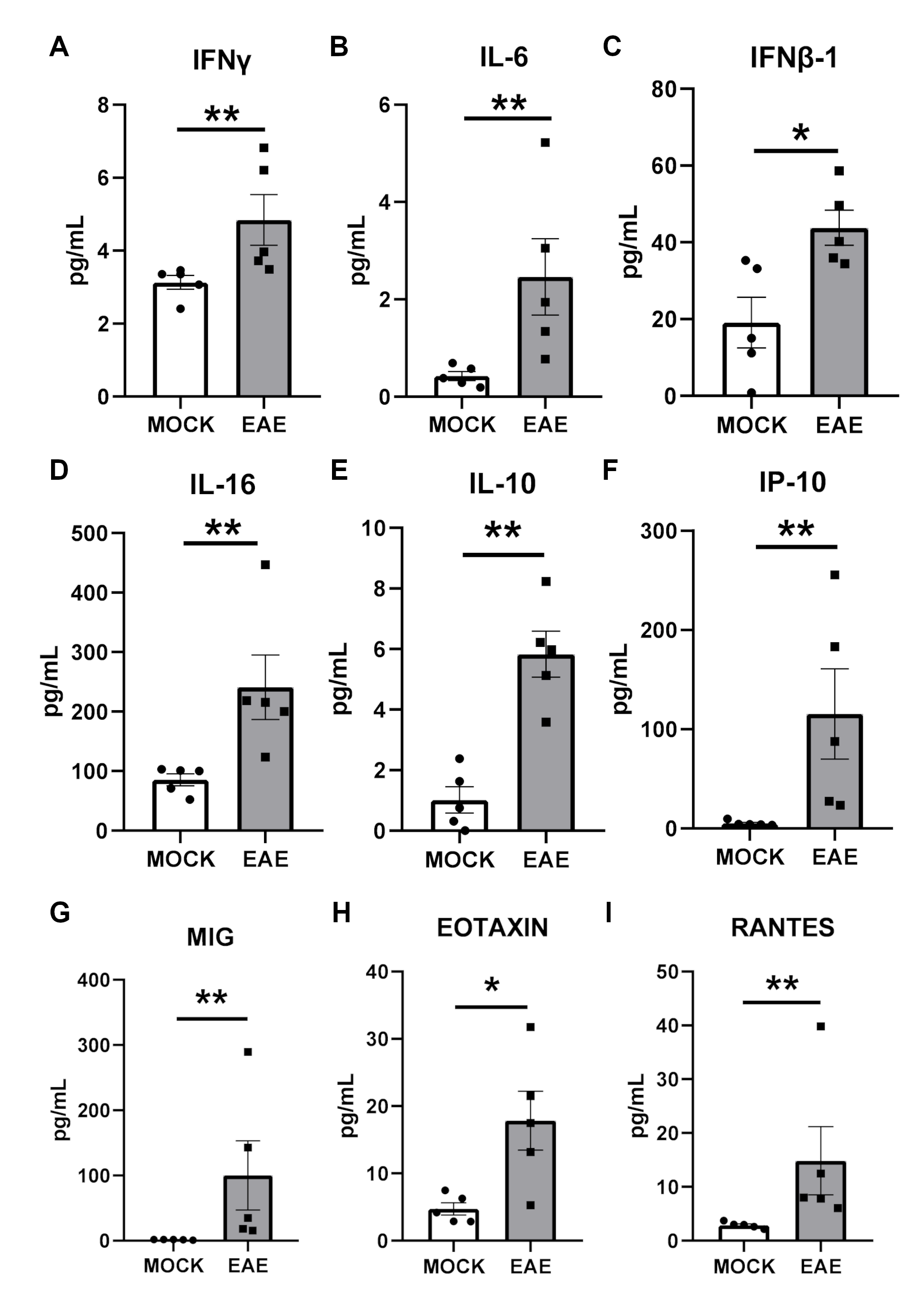

Supplement: Supplementary file 3 — Additional file 3: Figure S3. Dysregulated cytokines in the hypothalamus upon EAE. A–I Bar plots showing the concentration levels of interferon-gamma (IFN-γ), interleukin-6 (IL-6), interferon-beta (IFN-β), interleukin-16 (IL-16), interleukin-10 (IL-10), interferon gamma-induced protein 10 (IP-10), monokine induced by gamma (MIG), eotaxin, and regulated upon activation, normal T cell expressed and presumably secreted (RANTES) in the hypothalamus of EAE and control mice at 20 dpi. Data are plotted as means ±SEM (N = 5 per group from one EAE immunization) and differences between experimental groups were assessed by Mann-Whitney U-test. *P ≤ 0.05, **P ≤ 0.01; ***P ≤ 0.005. [file 12974_2023_3006_MOESM3_ESM.tif]

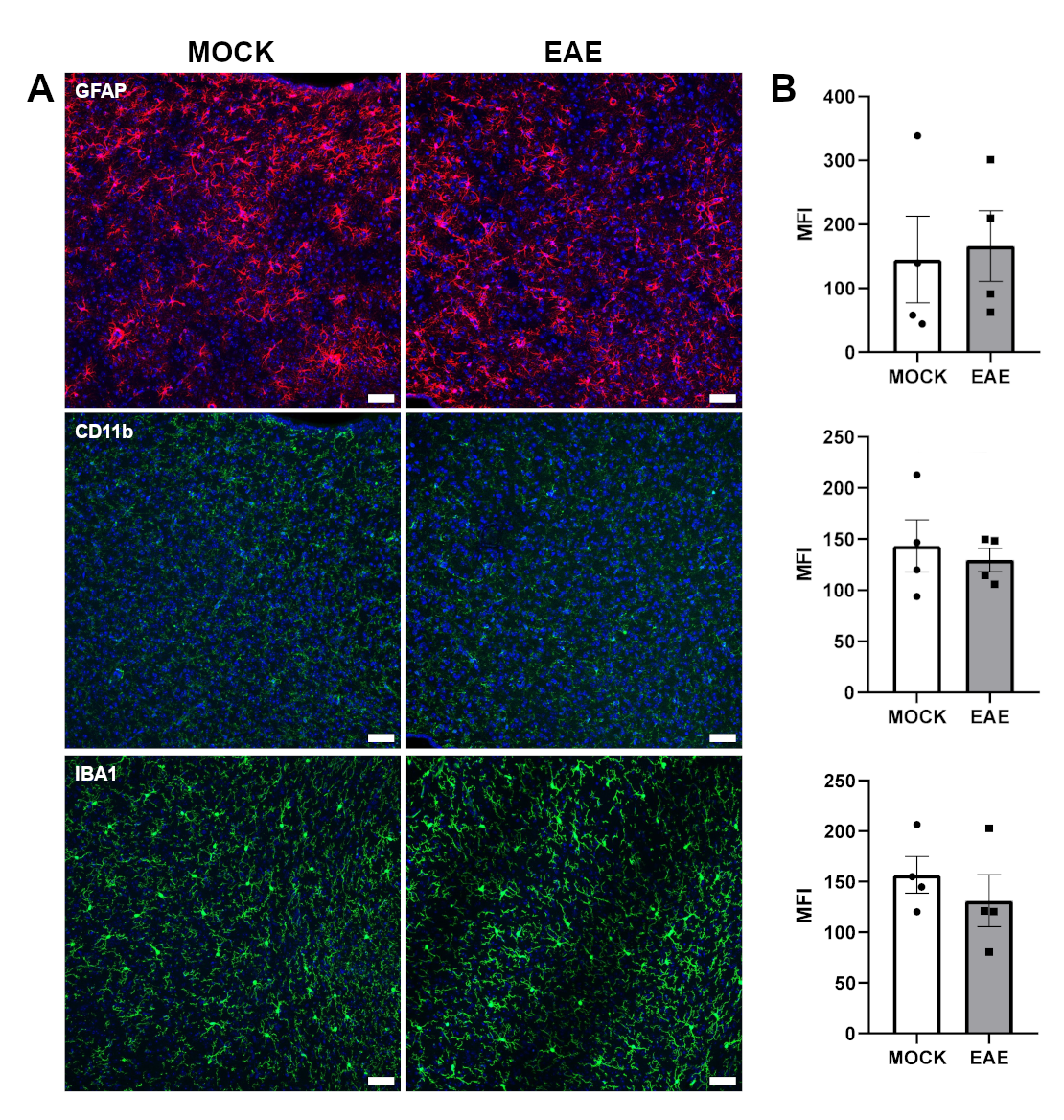

Supplement: Supplementary file 4 — Additional file 4: Figure S4. Analysis of reactive glia in the hypothalamus upon EAE. A Representative images of immunofluorescence staining for astrocytes (GFAP, red) and microglia (CD11b and IBA1, green) in the hypothalamus of EAE mice and controls at 20 dpi. Nuclei were counterstained with DAPI (blue). B Quantification of the different stains expressed as mean fluorescent intensity (MFI) values. Data are plotted as means ±SEM (N = 4 per group from one EAE immunization) and differences between experimental groups were assessed by Mann-Whitney U-test. Magnification = 10 ×, scale bar = 50 μm. [file 12974_2023_3006_MOESM4_ESM.tif]

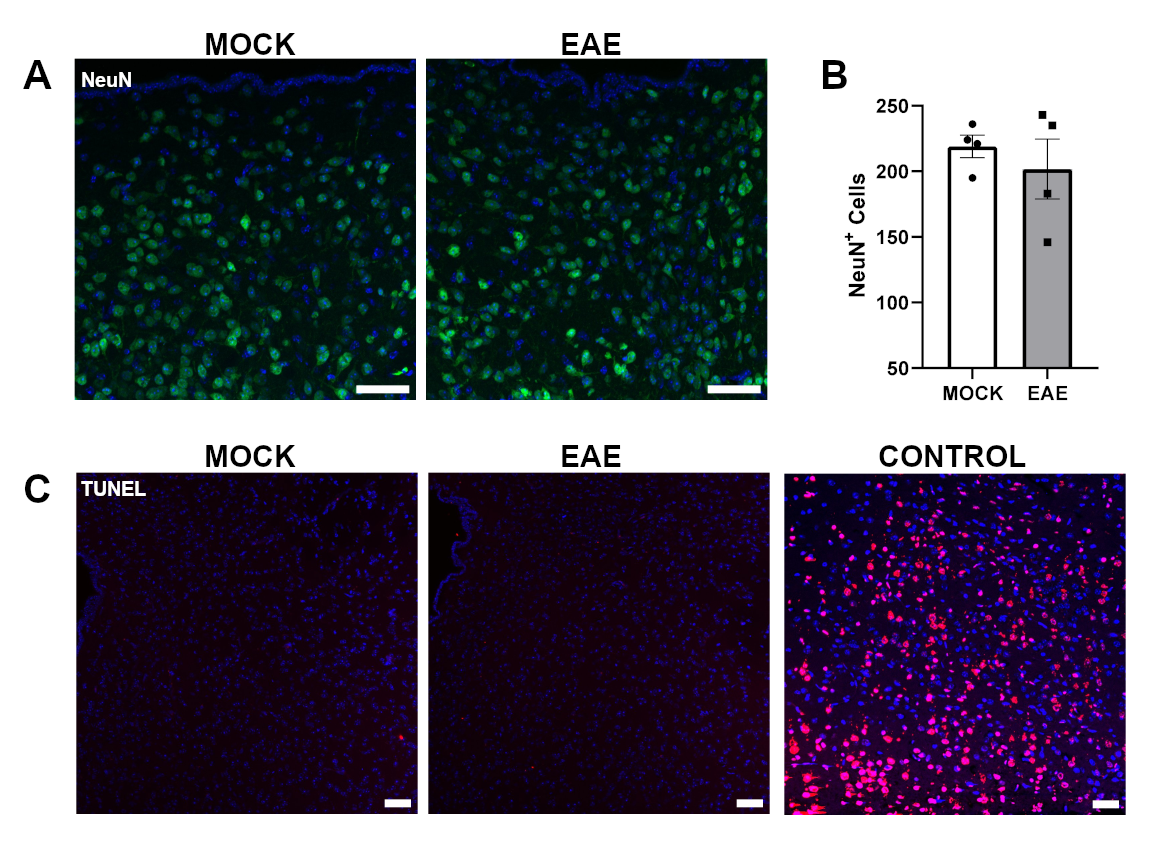

Supplement: Supplementary file 5 — Additional file 5: Figure S5. Analysis of neurodegeneration in the hypothalamus upon EAE. A Representative images of TUNEL staining (red) in the hypothalamus of EAE mice and controls at 20 dpi. Nuclei were counterstained with DAPI (blue). B Staining and relative quantification of neuronal cells (NeuN) in the hypothalamus at the same time point. Magnification = 20 ×, scale bar = 25 μm. C Representative images of TUNEL stained (red) hypothalamic sections from EAE and control mice at 20 dpi. As positive control, some sections were treated with DNase I. Nuclei were counterstained with DAPI (blue). Magnification = 10 ×, scale bar = 25 μm. Data are plotted as means ± SEM (N = 4 per group from one EAE immunization) and differences between experimental groups were assessed by Mann-Whitney U-test. [file 12974_2023_3006_MOESM5_ESM.tif]

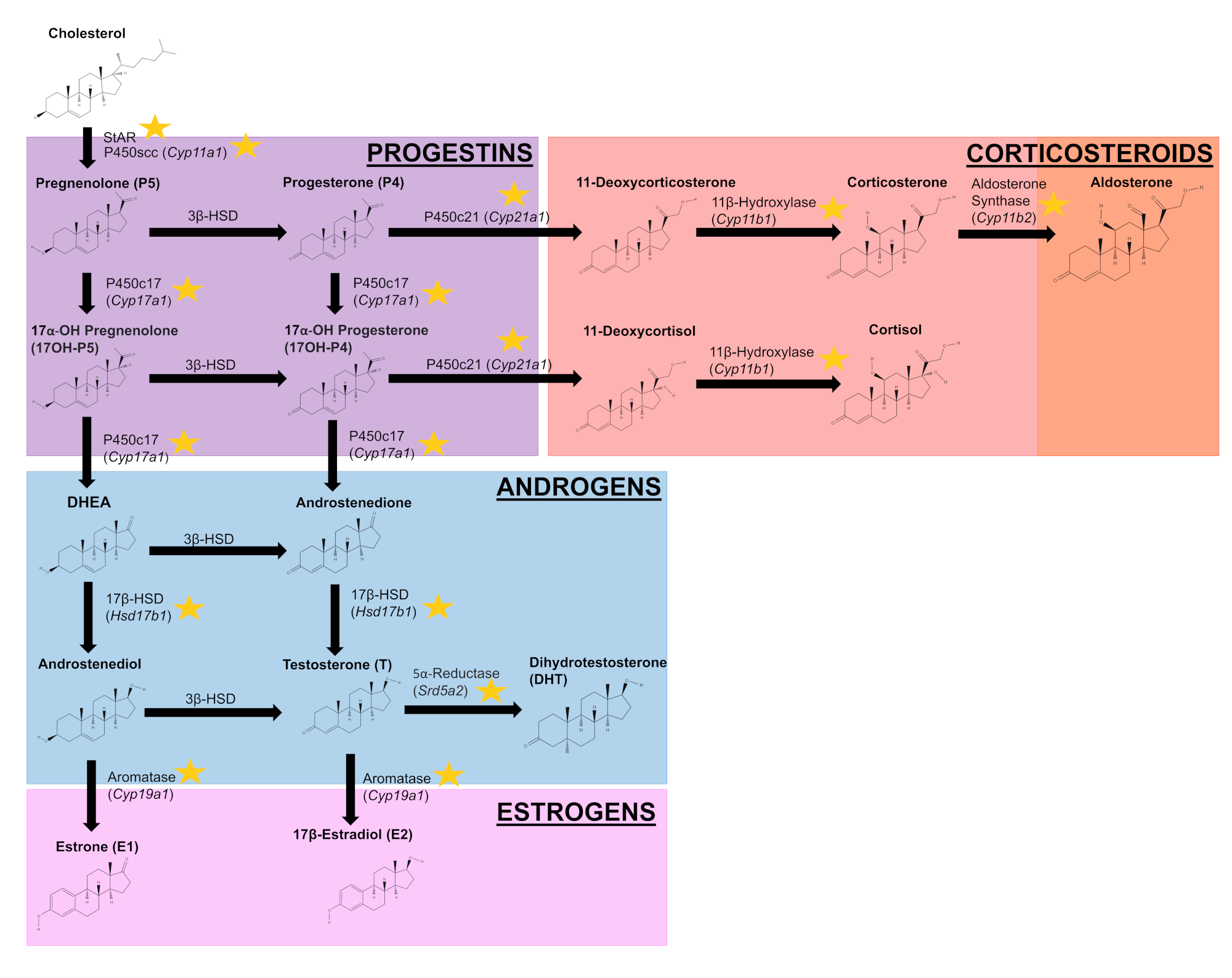

Supplement: Supplementary file 6 — Additional file 6: Figure S6. Target genes in the steroidogenesis pathway. A scheme of the main biosynthetic pathways for steroid hormones is depicted. Cholesterol is the common precursor for all the classes: progestins (purple), androgens (blue), estrogens (pink), glucocorticoids (red), and mineralocorticoids (orange). In female mice, corticosteroids and DHEA are mainly produced in the adrenal cortex layers. Estrogens, progestins, and some androgens are produced from ovarian follicles and corpora lutea in the ovary. Steroidogenic enzyme genes tested by qRT-PCR for differential expression between EAE and control animals are signified by a yellow star. Chemical structures were drawn using MolView software. [file 12974_2023_3006_MOESM6_ESM.tif]

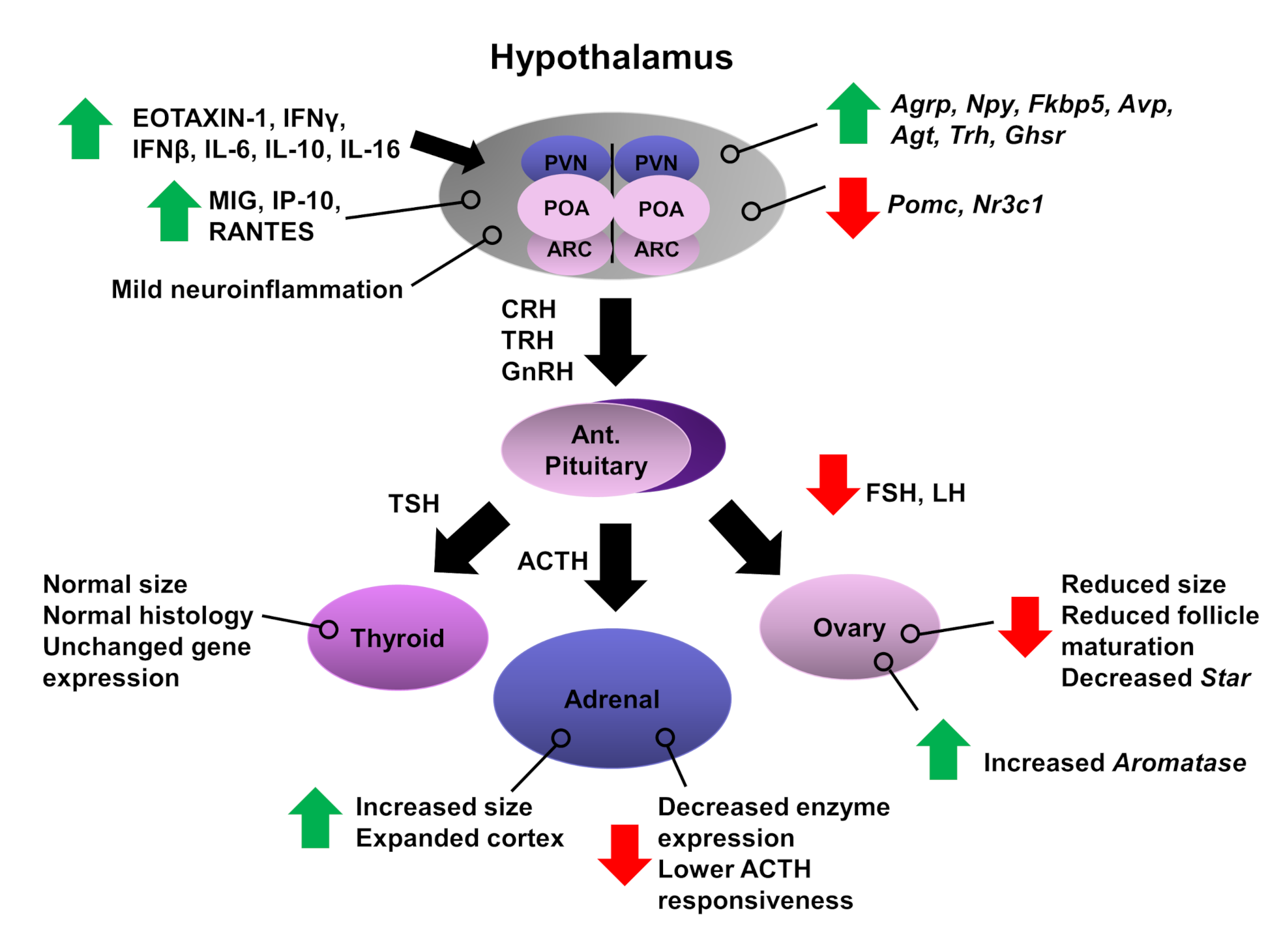

Supplement: Supplementary file 7 — Additional file 7: Figure S7. Proposed working model of hypothalamic dysfunction in autoimmune demyelination. EAE pathology specifically leads to an increase of specific cytokines (CCL-11, IFNγ, IFNβ, IL-6, IL-10, and IL-16, MIG, IP-10, and RANTES) in in the absence of local lesions or reactive glial species in the hypothalamus. At the same time, several neuropeptides involved in maintaining the physiological homeostasis are differentially expressed, including Agrp, Npy, Avp, Agt, Pomc, Trh, glucocorticoid receptor Nr3c1 and its cochaperone Fkbp5. The hypothalamus contains pulse generator neurons for the HPT/HPA axes in the paraventricular nucleus (PVN), and HPG axis in the arcuate nucleus (ARC) and preoptic area (POA). During acute EAE, the adrenal gland is significantly enlarged with decreased expression of steroidogenic enzymes, suggesting chronic overstimulation of HPA axis and gaining ACTH resistance. Conversely, the ovaries are significantly smaller with fewer viable follicles and corpora lutea, likely due to lower levels of both gonadotropins FSH and LH. The overexpression of Cyp19a1 may represent a homeostatic response to maintain estrogen production. [file 12974_2023_3006_MOESM7_ESM.tif]
